# Non-Invasive Parameter Identification of DC Arc Models for MV Circuit Breaker Diagnostics

**DOI:** 10.3390/s25103161

**Published:** 2025-05-17

**Authors:** Gabriele D’Antona, Camilo Trujillo-Arboleda, Massimiliano Amato, Marco Riva

**Affiliations:** 1 Department of Energy, Politecnico di Milano, 20156 Milan, Italy; camilo.trujillo@polimi.it; 2 ELDS Technology Center, ABB S.p.A., 24044 Bergamo, Italy; massimiliano.amato@it.abb.com (M.A.); marco.riva@it.abb.com (M.R.)

**Keywords:** DC arc model, DC circuit breaker, arc voltage, parameter estimation, Kalman filter, data assimilation

## Abstract

Accurate electrical arc modeling with physically meaningful parameters is essential for the assessment of medium-voltage DC circuit breakers in industrial and railway applications. Laboratory testing and characterization, as outlined in the IEC 61992 standard series for railway applications, typically provide data to asses the operational behavior of the componentsin the power distribution system, including recorded waveforms of terminal voltage and current but not the insights and inputs needed for inner behavior analysis and design optimization. This paper introduces lumped-parameter multi-physics models to describe different phases of arc behavior and outlines a methodology for model–data assimilation. Using experimental test data, the approach enables performance evaluation and supports non-invasive diagnostics and potential condition monitoring of circuit breakers.

## 1. Introduction

Medium-voltage DC switch breakers are employed across a wide range of sectors where the reliable interruption and control of DC currents are critical. Key application areas include railway systems, marine and offshore platforms, renewable energy and energy storage systems, industrial and mining operations, microgrids and smart grids, electric vehicle infrastructure, aerospace and defense, data centers and high-energy physics and nuclear fusion experiments.

Interrupting direct current (DC) presents several critical challenges, primarily due to the continuous nature of the current flow. One major issue is the formation of high-voltage arcs when the circuit is open, which can cause significant damage to electrical components. Rapid disconnection is essential to prevent overheating and equipment failure, but achieving this without compromising the stability of the system is complex.

Air Arc-chute Circuit Breakers (AACBs) offer a solution by using air as the medium to extinguish the arc. These breakers are designed to quickly separate contacts within an air chamber, where the arc is stretched and cooled until it is extinguished. This method is effective in managing the high energy levels associated with DC systems, ensuring safety and reliability. However, the design and maintenance of AACBs must be meticulously managed to ensure proper functioning under all operating conditions.

The formation and evolution of an electric arc inside the extinguishing chamber of switch breakers is a complex phenomenon. It can be modeled by employing finite-element numerical solvers to capture the multi-physics aspects and the spatial distribution of the physical quantities involved. Despite their complexity, these models are invaluable for understanding the phenomena and optimizing breaker switch designs. In the comprehensive analysis presented by Gordon [1], the author dives into the historical evolution and current state of DC arc modeling. The discussion spotlights the predictive inadequacies of static models, where many arc parameters remain constant over time, and the significance of dynamic models, which incorporate theoretical dependencies such as convective forces, variations in arc length and the influence of magnetic forces.

However, while these models provide detailed spatial and temporal insights into electrical discharges, they rely on numerous parameters that are often unknown, unavailable and not easily measured by standard test procedures. In contrast, simpler black-box models offer a more accessible approach by employing simple arc behavior models. For example, the classical Mayr model combines electrical and thermodynamic modeling, representing the arc as a rigid volume with uniform temperature and electrical temperature-dependent conductance. The temperature evolution is driven by the heating power, which is assumed to be the product of the arc current and voltage, while the cooling power influences the unknown arc’s thermal resistance and time constant. This model eventually leads to expression of the arc’s electrical conductance as a nonlinear differential equation that depends just on three unknown constant parameters, including the arc’s initial conductance.

More advanced black-box arc models (see, for example [2,3,4]) represent the arc plasma column as an apparent conductance, without incorporating key physical effects but considering different phases in the arc’s evolution:

- Arc movement and elongation along the arc rails;
- Inductive effects influencing the measured voltage drop at the switch breaker terminals;
- Voltage drops at the arc roots in the splitter plates (SPs);
- Heat transfer dynamics between the arc, the surrounding insulating medium and the splitter plates.

Black-box models are widely used in circuit simulations to explore electrical transients in the system during circuit breaker operations, but they provide only a limited understanding of the internal physics of the switch breakers’ electrical arc. A comprehensive analysis of the assumptions and limitations of a wide range of black-box arc models, including the classical Mayr model, can be found in [5].

Additionally, these models have historically been developed for AC arcs, limiting their direct applicability to DC systems. Examples of the implementation of black-box models during arc interruption with DC circuit breakers can be found in [6,7,8].

**Figure 1:**
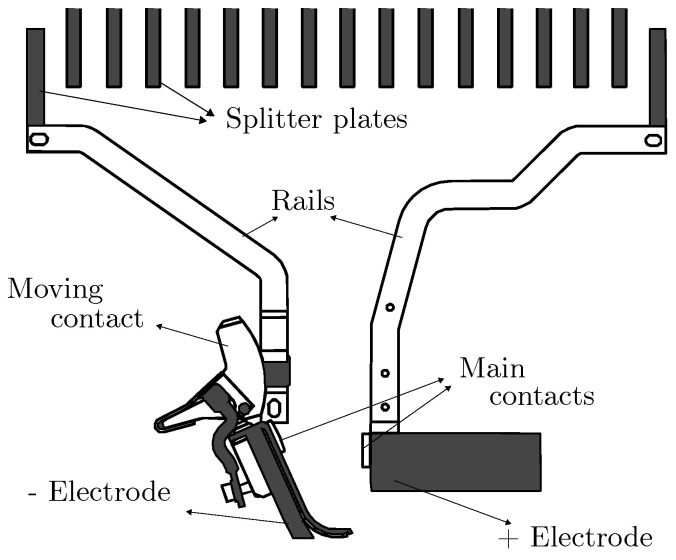
Typical internal construction of an MV air–insulated DC circuit breaker.

The behavior of arc voltage and current in DC circuit breakers differs significantly from that in AC systems. In DC interruption, the arc is extinguished only when its voltage rises above the network voltage, forcing the current to zero. Unlike AC arcs, which naturally extinguish at current zero crossings, DC circuit breakers must be designed to undergo multiple transitional phases before current interruption occurs, especially when using air-insulated technology. Figure 1 shows the main components of the arc chamber in industrial air-insulated DC circuit breakers. The key phases of a DC arc evolution are listed as follows

1. *Contacts Closed:* The circuit breaker is closed, and the circuit is energized. The current is flowing, and no arc is present.
2. *Contact Opening:* As the contacts start to separate, an arc forms due to the breakdown of the dielectric medium. Current still flows in the circuit.
3. *Contacts Opened—Arc on Rails:* The arc is transferred to arc runner rails, where it elongates due to electromagnetic and aerodynamic forces. Current still flows in the circuit.
4. *Contacts Opened—Arc Inside Splitter Plates:* The arc enters the splitter plate stack, where it undergoes cooling, an increase in voltage and eventual extinction.

To accurately capture these complexities, this paper introduces a set of multi-physics arc models specifically tailored for DC interrupters in air, such as those illustrated in Figure 1. Although simplified, the models account for the following:

1. The flow of electrical current imposed by the laboratory test setup according to relative product standards;
2. The mechanical dynamics of the arc’s movement;
3. The thermal evolution of the arc.

These models are lumped parameter models, meaning they are characterized by a finite number of state variables (degrees of freedom). Unlike purely empirical black-box models, this approach integrates available test data, such as the laboratory test circuit and the measured time evolution of voltage and current at the switch breaker terminals, with the arc models. By assimilating these data, the model estimates the internal state variables of the arc, providing insight into the physical state of the device under test. This method aims to characterize the internal state of the AACB, offering both laboratory-based assessments and potential applications for real-time monitoring in electrical systems.

The remainder of this paper is organized as follows: Section 2 presents the air arc chute technology and the prototype used for experimental and model calibration. Section 3 presents the physical models that describe the evolution of the arc inside the circuit breaker’s extinguishing chamber. In Section 4, a recursive estimation approach based on a Kalman filter is introduced to estimate the state variables of the models. Section 5 presents the results obtained from experimental laboratory data for a prototype of a representative circuit breaker and a comparison with traditional models from the literature. Finally, Section 6 summarizes the main conclusions, highlights the limitations of the proposed approach and identifies opportunities for future research.

## 2. DC Circuit Breaker

### 2.1. Air Arc Chute Circuit Breakers

**Figure 2:**
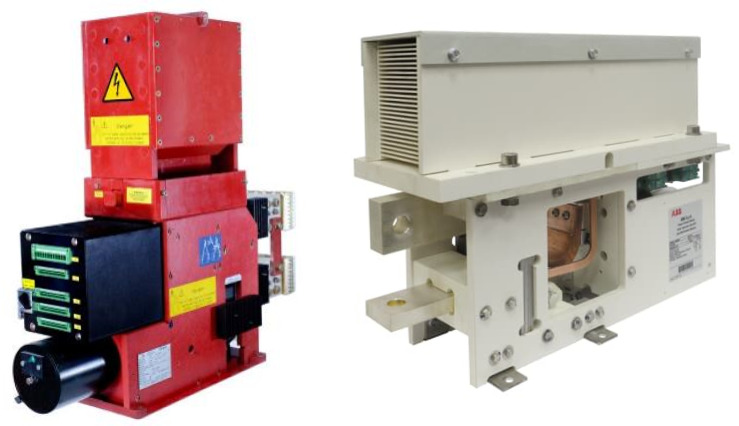
Images of Gerapid (**left**) and DCBreak (**right**) ABB high-speed DC circuit breakers.

Single-pole, high-speed DC air circuit breakers based on air arc chute technology are widely used in railway and industrial applications. The high-speed feature allows for the safe interruption and clearing of high prospective short-circuit peaks in a few tens of milliseconds. This portfolio of DC circuit breakers is suitable for feeder, rectifier or rolling stock protection devices in high power, low- and medium-voltage DC distribution systems (1500–8000 A and 800–3600 V), covering IEC, EN and ANSI requirements [9,10,11]. Figure 2 reports typical DC circuit breakers used in railway applications.

The apparatus considered to calibrate and test the model is a prototype AACB and consists of the following main components:

- A contact system equipped with main and arcing contacts and a double-contact design, providing high wear-resistance under arc erosion;
- A mechanism to ensure the closing and opening of the contacts by means of manual mechanical operation or connected closing/tripping devices, designed to guarantee extended electrical and mechanical endurance;
- An arc chute consisting of arc plates to split the electric arc generated by current interruption into smaller partial arcs with a subsequent increase in arcing voltage;
- A solenoid closing drive to enable closing of the main contacts, designed to avoid repeated or premature closing operations during an existing short-circuit event, increasing the protection level;
- Additional tripping devices to enable the de-latching mechanism and subsequent contact opening. Tripping devices can be designed for “slow” opening operations (such as remotely driven actuation or actuation driven by under-voltage release) or for “quick” opening operations in the case of more severe faults such as overload and short circuits. In the prototype used for this paper, the overload tripping device consists of a ferromagnetic core able to sense the current fault level and directly trip the AACB mechanism;
- An electronic control system, including several electronic units, mainly to control the solenoid closing drive and tripping functionalities;
- Auxiliaries and accessories for additional features related to signaling, tripping and temporary blocking of the mechanism.

A DC circuit breaker prototype was used to build the current model. Short-circuit experimental data obtained in dedicated tests represent a solid base to properly analyze current interruption performances and to refine the presented model parameters.

### 2.2. Short-Circuit Test Setup

Short-circuit interruption is one of the most critical performance areas required in a circuit breaker. Current breaking must occur as quickly as possible and before that fault level reaches high peak values that could compromise the functioning of infrastructure or human health. As previously mentioned, DC current interruption is more challenging than in AC networks, since no natural zero-current point exists to assist in fault breaking. To successfully limit and interrupt peak currents, a DC current breaker must properly guide the electric arc from separating contacts to the arc chute and stretch it to generate a sufficient increase in arc voltage. When the arc voltage exceeds the sum of the source voltage and load voltage drop, the current starts to abruptly decrease until it is fully interrupted.

**Figure 3:**
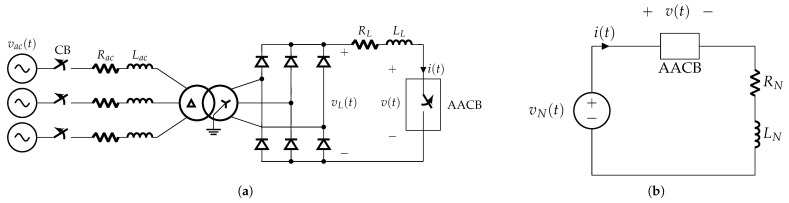
Short-circuit test configuration: (**a**) laboratory circuit; (**b**) equivalent circuit.

The simplified circuit used in the experimental tests presented in Figure 3a can be further reduced to an equivalent form [12], as shown in Figure 3b. The governing electrical relationship for this equivalent circuit is expressed as

(1) $$v_{N}=L_{N}\frac{di}{dt}+R_{N}i+v\;,$$

where $v_{N}$ represents the equivalent supply voltage; $L_{N}$ and $R_{N}$ denote the equivalent inductance and resistance at the AACB terminals, respectively; *v* is the voltage drop across the AACB, primarily due to the contribution of the arc voltage; and *i* is the current flowing through it. Rearranging (1) yields

(2) $$L_{N}\frac{di}{dt}=v_{N}-R_{N}i-v\;.$$

This equation highlights that, in order to successfully interrupt the circuit (bringing the current to zero) and extinguish the arc, the condition of di/dt<0 must be met. This requires the arc voltage build-up within the AACB to be sufficiently large to counteract both the supply voltage and the voltage drop across the circuit resistance.

**Figure 4:**
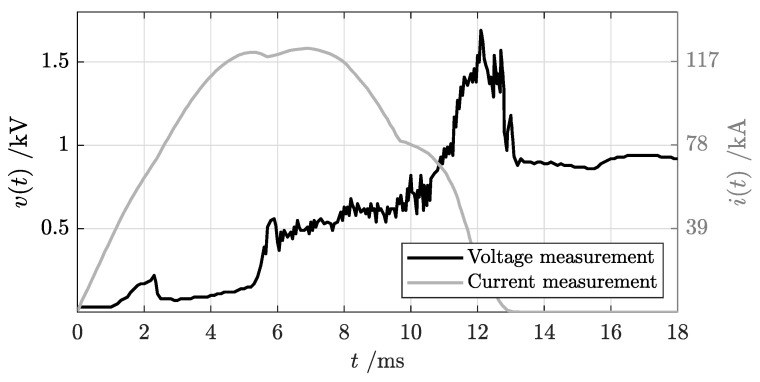
Representative oscillogram from an AACB showing the voltage (v(t)) and current (i(t)) measured at the circuit breaker terminals during a short−circuit test.

A typical oscillogram from the short-circuit test includes voltage–current profiles over time measured at the terminals of the circuit breaker, as shown in Figure 4. Let us suppose that the fault occurs at time $t_{0}$, with a prospective current ($I_{cn}$) rising with a di/dt ratio that depends on the time constant ($\tau =L_{N}/R_{N}$) of the circuit. In case of a fault, an initial time interval is required by the protection system to detect and react to the anomalous overcurrent/voltage and to operate the mechanism to begin opening contacts. At this instant, an electric arc starts to form and is pushed to the arc chute, leading to an increase in arc voltage. In the meantime, the fault current continues to increase until the arc resistance introduced in the circuit is enough to force the current to drop and to be fully interrupted. This gradual extinction of short-circuit current is accomplished by stretching the arc and splitting it into smaller partial arcs by means of the arc chute. Since no current zero points are present in a DC fault, current breaking is guaranteed only if the arc voltage (*v*) remains greater than the supply voltage ($v_{N}$) minus the resistive voltage drop, as in Equation (2).

The EN standard [9] prescribes that the short test procedure be performed on high-speed DC circuit breakers for railway applications. Different fault cases are depicted depending on the values of the impedance of the circuit ($L_{N},\;R_{N},\;\tau \;=\;L_{N}/R_{N})$, which represent distinct locations of the fault along the rail distribution line.

**Figure 5:**
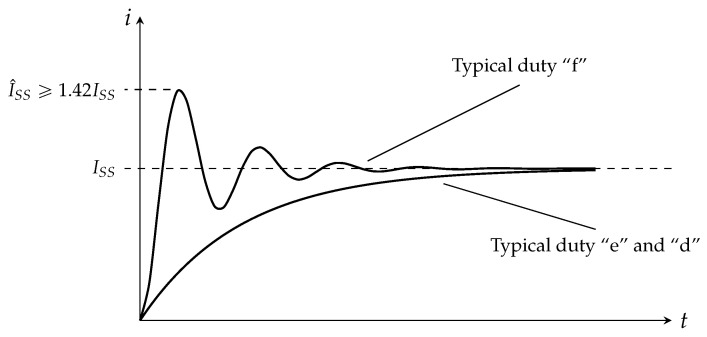
Breaking current parameters for duty tests.

The typical current profile generated by the fault test sequence according to [9] is shown in Figure 5. $I_{ss}$ is defined as the prospective sustained short-circuit current with a peak of ${\hat{I}}_{ss}$. The three test duties “f”, “e”, and “d” as defined in [9] are described below:

- Duty “f” represents the case of a fault occurring close to a substation where impedance is negligible. The prospective short-circuit current reaches the maximum fault level with a peak of ${\hat{I}}_{ss}\geq 1.42I_{ss}$.
- Duty “e” is the maximum circuit energy fault: as the short-circuit occurs at a longer distance from the substation, the circuit time constant (τ) gradually moves from the value at the source to the value of the track. When the circuit time constant is approximately in the middle of these two extreme values, the maximum-energy fault occurs. According to the rated track time constant declared for the circuit breaker, the standard prescribes the test circuit parameters.
- Duty “d” represents a distant short-circuit fault. Test parameters include a current value typically twice the nominal-load current of the breaker and a circuit time constant equal to the rated track-time constant of the AACB.

The highest current duty (“f”) shows a steeper initial current rise (di/dt) compared to the sharper exponential increase of duties “d” and “e” as a result of differences in circuit time constant values.

Each of the duty tests prescribed by the EN standard [9] consists of a sequence of O-CO-CO-CO operations (where O stands for opening and CO stands for closing–opening) with a time interval of 15 s between each shot. For modeling purposes, only a CO is considered for oscillogram comparison with simulated data. This choice was made to achieve a wider statistical base compared to a single O operation for each duty and to consequently reduce model uncertainties.

## 3. Modeling the DC Apparatus: The Thermo-Electrical Approach

As mentioned Section 1, the evolution of a DC arc can be divided into four key phases: *contacts closed*, where no arc is present; *contact opening*, leading to arc formation; *arc on rails*, during which the arc elongates; and *arc inside splitter plates*, where the arc is further cooled and the voltage of the multiple arc roots counteracts the applied voltage.

The models describing each phase (*j*) of the arc’s evolution are formulated as continuous-time dynamical systems, represented by a set of nonlinear differential equations:

(3) $$\left\{\begin{array}{cc} {\dot{x}}_{j}\left(t\right)=f_{j}\left(x_{j}\left(t\right),v_{N}\left(t\right)\right)+e_{j}\left(t\right),\; & Q_{j}^{\prime }\left(t\right)=\mathrm{cov}\left\{e_{j}\left(t\right)\right\}\\ y\left(t\right)=h_{j}\left(x_{j}\left(t\right),v_{N}\left(t\right)\right)+\epsilon_{j}\left(t\right),\; & R_{j}^{\prime }\left(t\right)=\mathrm{cov}\left\{\epsilon_{j}\left(t\right)\right\} \end{array}\right.$$

In these equations,

- $x_{j}\left(t\right)$ represents the state vector of the *j*-th model;
- $f_{j}\left(\;\cdot \;\right)$ dNefines the system dynamics;
- $v_{N}(t)$ is the supply voltage, serving as the input to every model;
- y(t) consists of the measured quantities, which, for each model, include the measured voltage and current of the AACB;
- $h_{j}\left(x_{j},v_{N}\right)=\;m$ represents the vector measurands, expressed as a function of the *j*-th model state and input;
- $e_{j}\left(t\right)$ and ϵ(t) denote stochastic processes modeling system and measurement errors, with covariance matrices $Q_{j}^{\prime }\left(t\right)$ and $R_{j}^{\prime }\left(t\right)$, respectively.

For every model, the measurands consist of the instantaneous values of the measured voltage (v(t)) and current (i(t)) of the AACB, which are organized into the measurand vector (m) as follows:

(4) $$m={\left[\begin{array}{cc} i & v \end{array}\right]}^{T}$$

The continuous-time models in (3) are discretized using the backward Euler approximation for the time derivative [13], yielding the following finite-difference equations for each model phase:

(5) $$\left\{\begin{array}{c} x_{j}\left(k\right)=x_{j}\left(k-1\right)+\Delta t_{S}\;\cdot \;f_{j}\left(x_{j}\left(k-1\right),v_{N}\left(k-1\right)\right)+\Delta t_{S}\;\cdot \;e_{j}\left(k-1\right)\\ y\left(k\right)=h_{j}\left(x_{j}\left(k\right),v_{N}\left(k\right)\right)+\epsilon_{j}\left(k\right) \end{array}\right.$$

where $\Delta t_{S}$ represents the sampling period and the state vector ($x_{j}\left(k\right))$ is updated at each discrete time step (*k*) based on the previous state ($x_{j}\left(k-1\right)$). The covariance matrices of the discrete-time process and measurement errors are expressed as

(6) $$\left\{\begin{array}{c} Q_{j}\left(k\right)=\mathrm{cov}\left\{\Delta t_{S}\;\cdot \;e_{j}\left(k\right)\right\}=\Delta t_{S}^{2}\;\cdot \;Q_{j}^{\prime }\left(k\right)\\ R_{j}\left(k\right)=\mathrm{cov}\left\{\epsilon_{j}\left(k\right)\right\}=R_{j}^{\prime }\left(k\right) \end{array}\right.$$

The following sections introduce the specific models proposed to describe each phase of the arc’s evolution.

### 3.1. Phase 1: Contacts Closed

**Figure 6:**
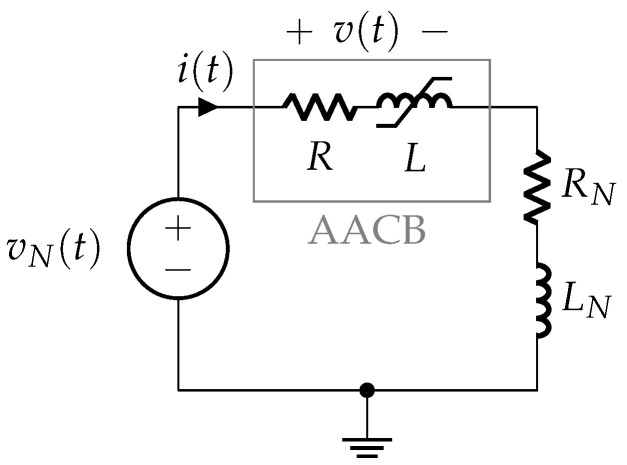
Equivalent electrical circuit during the closed-contact phase.

The simplified electrical circuit describing this phase is shown in Figure 6. The AACB is supplied by voltage ($v_{N}\left(t\right)$) from the laboratory rectifier voltage source, and the current flowing in the circuit is limited by the series connection of the resistance (*R*) and the inductance (*L*) within the AACB’s internal current path, alongside the resistance ($R_{N}$) and inductance ($L_{N}$) specified by the laboratory setup to generate the correct prospective current profile.

The internal resistance (*R*) is approximated as the contact resistance, assuming the electrodes’ resistance is negligible (see Figure 1). The internal inductance (*L*) of the AACB is nonlinear, as it depends on the iron core of the magnetic protection relay, which controls the AACB overcurrent tripping mechanism, as described in Section 2.1. During this phase, the circuit current i(t) is mainly dependent on the resistance ($R_{N}$) and inductance ($L_{N}$). The voltage (v(t)) in Figure 6 represents the terminal voltage of the AACB.

The thermal heating during this phase can be considered negligible, as demonstrated by a simple adiabatic heating analysis of the contacts. Considering a contact resistance (*R*) of approximately 10 μΩ and a maximum AACB fault current of *I* around 30 kA, the dissipated heat power is estimated as

$$q\;=\;RI^{2}\;=\;9\;kW$$

Assuming the contact material is silver zinc oxide with a volume (*V*) of approximately 3 cm^3^, the heat capacity of the contacts ($C_{C}$) is around 6 J/K. Considering that the contacts remain closed for a duration of Δt = 1 ms and assuming adiabatic heating, the resulting temperature increase is

$$\Delta T\;=\;q\Delta t/C_{C}=\;1.5\;K$$

This temperature rise is negligible for the target accuracy of the physical model discussed in this paper, especially when considering the high temperatures reached by the arc and surrounding materials during the subsequent arc model phases, which are discussed next.

Assume that the following data are available, along with their associated uncertainties:

- The electrical parameters of the laboratory set-up: resistance ($R_{N}$) and inductance ($L_{N}$), as well as the supplying voltage ($v_{N}\left(t\right)$);
- The inductance (*L*) as a function of the current (i(t)).

Also assume that the resistance (*R*) is modeled as an unknown, approximately constant quantity. Therefore, the equations governing the system are

(7) $$\left\{\begin{array}{cc} v_{N}=\left(R+R_{N}\right)i+\left(L+i\frac{dL}{di}+L_{N}\right)\frac{di}{dt}\;, & i(t_{1})=0\\ \frac{dR}{dt}=0\;, & R\left(t_{1}\right)=\bar{R} \end{array}\right.\;.$$

where time instant $t_{1}$ represents the initial time in the phase 1 model. The non-zero initial conditions in (7) include an guessed initial value for the contact resistance (*R*).

The model equations in (7) are used to derive the equations in the form of (3) when the stochastic error term ($e_{1}\left(t\right)$) is included, which quantifies the approximation provided by the model.

The model described by (7) involves two state variables. A suitable choice for the state vector is expressed as

(8) $$x_{1}={\left[\begin{array}{cc} i & R \end{array}\right]}^{T}\;.$$

In accordance with (3), during the closed-contact phase, the AACB model (7) can be reformulated as

(9) $$\frac{dx_{1}}{dt}=f_{1}\left(x_{1},v_{N}\right)+e_{1}\left(t\right),\;\mathrm{initial}\;\mathrm{condition}\;\mathrm{as}\;\mathrm{in}\;\left(7\right)\;$$

Considering Equation (7) and the expression for the AACB voltage (see Figure 6), i.e.,

(10) $$v=Ri+\left(L+i\frac{dL}{di}\right)\frac{di}{dt},$$

the measurands (4) can be expressed in terms of the state vector ($x_{1}$) and the driving voltage ($v_{N}$) through the following function vector:

(11) $$h_{1}\left(x_{1},v_{N}\right)=\left[\begin{array}{c} x_{1}\left[1\right]\\ \frac{L_{N}x_{1}\left[2\right]-\left(L+x_{1}\left[1\right]\frac{dL}{di}\right)R_{N}}{L+x_{1}\left[1\right]\frac{dL}{di}+L_{N}}x_{1}\left[1\right]+\frac{L+x_{1}\left[1\right]\frac{dL}{di}}{L+x_{1}\left[1\right]\frac{dL}{di}+L_{N}}v_{N} \end{array}\right]$$

where square-bracket notation $x_{1}\left[m\right]$ denotes the *m*-th element of state vector $x_{1}$. To improve readability, explicit time dependencies are omitted in the equation.

### 3.2. Phase 2: Contact Opening

During the opening phase of the contacts, the AACB model consists of both electrical and thermal components. A notable difference relative to previous models is the initiation of an arc plasma between the opening contacts.

**Figure 7:**
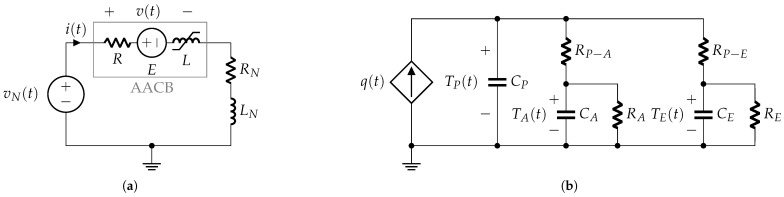
Model of the contact-opening phase: (**a**) electrical equivalent circuit; (**b**) thermal equivalent circuit.

The electrical model shown in Figure 7a differs from that of the previous phase, as the internal resistance (*R*) no longer represents the contact resistance but is now primarily determined by the higher resistance of the arc plasma.

The plasma resistance is highly sensitive to the uncertain position of the AACB contacts, whose time evolution can be complex to model due to contact bouncing. Consequently, the plasma resistance is approximated as an unknown constant. As previously mentioned, this model is subject to errors encapsulated in the stochastic term ($e_{2}\left(t\right)$), which accounts for unmodeled contact dynamics.

Additionally, a constant internal electromotive force (*E*) is introduced to represent the constant voltage drops at the arc roots on each contact, which is estimated to be around 20 V [14].

The thermal-electrical network shown in Figure 7b represents the lumped-parameter thermal dynamics [15] of the AACB during contact opening. This model consists of a heat power source, q(t), in parallel with the heat capacity $C_{P}$ of the electrical arc plasma. Heat transfers occur from the plasma to the surrounding air and electrodes.

In this representation, thermal resistances $R_{P-A}$ and $R_{P-E}$ (shown in Figure 7b) characterize thermal conduction between the plasma and air, and between the plasma and electrodes, respectively. Thermal capacities $C_{A}$ and $C_{E}$ denote the heat capacities of the surrounding air and electrodes. Additionally, the thermal resistances $R_{A}$ and $R_{E}$ connect the two heat capacities, modeling the thermal bridges between the external environment and the arc chamber air and electrodes, respectively.

Applying the thermal–electrical analogy, temperatures $T_{P}\left(t\right),\;T_{A}\left(t\right)$ and $T_{E}\left(t\right)$ represent the temperature rise (i.e., the excess temperature) of the plasma, air and electrodes relative to the environmental temperature, respectively. The heat power dissipated in the plasma (q(t)) is expressed as

(12) $$q\left(t\right)=R\;\cdot \;i{\left(t\right)}^{2}+E\;\cdot \;i\left(t\right)\;,$$

and, in the thermal–electrical analogy, is represented by a current source.

Assume that the following data are available, along with their associated uncertainties:

- The values of the external resistance ($R_{N}$) and inductance ($L_{N}$), as well as the supplying voltage ($v_{N}\left(t\right)$) (as in any phase model);
- The inductance (*L*) as a function of the current (i(t)) (as in the phase 1 model);
- The electromotive force (*E*);
- The thermal capacities of plasma, air and electrodes ($C_{P},\;C_{A}$ and $C_{E}$, respectively).

Also assume that the plasma resistance (*R*) and the thermal resistances ($R_{P-A}$ and $R_{P-E}$) are modeled as unknown, approximately constant quantities. Therefore, the equations governing the system are

(13) $$\left\{\begin{array}{cc} v_{N}=E+\left(R+R_{N}\right)i+\left(L+i\frac{dL}{di}+L_{N}\right)\frac{di}{dt}\;, & i\left(t_{2}\right)=i\left(t_{2}^{-}\right)\\ \frac{dR}{dt}=0\;, & R\left(t_{2}\right)=R\left(t_{2}^{-}\right)\\ Ri^{2}+Ei=C_{P}\frac{dT_{P}}{dt}+C_{A}\frac{dT_{A}}{dt}+\frac{T_{A}}{R_{A}}+C_{E}\frac{dT_{E}}{dt}+\frac{T_{E}}{R_{E}}\;, & T_{P}\left(t_{2}\right)=T_{A}\left(t_{2}\right)=T_{E}\left(t_{2}\right)=0\\ C_{A}\frac{dT_{A}}{dt}+\frac{T_{A}}{R_{A}}=\frac{T_{P}-T_{A}}{R_{P-A}}\;, & T_{A}\left(t_{2}\right)=0\\ C_{E}\frac{dT_{E}}{dt}+\frac{T_{E}}{R_{E}}=\frac{T_{P}-T_{E}}{R_{P-E}}\;, & T_{E}\left(t_{2}\right)=0\\ \frac{dR_{P-A}}{dt}=0\;, & R_{P-A}\left(t_{2}\right)={\bar{R}}_{P-A}\\ \frac{dR_{A}}{dt}=0\;, & R_{A}\left(t_{2}\right)={\bar{R}}_{A}\\ \frac{dR_{P-E}}{dt}=0\;, & R_{P-E}\left(t_{2}\right)={\bar{R}}_{P-E}\\ \frac{dR_{E}}{dt}=0\;, & R_{E}\left(t_{2}\right)={\bar{R}}_{E} \end{array}\right.\;.$$

were time instant $t_{2}$ represents the initial time in the phase 2 model. Initial conditions are determined by guessed values for the thermal resistances ($R_{P-A},\;R_{A}$ and $R_{P-E},R_{E}$). The initial temperature rises of the plasma, air and electrodes are set to zero, as heating during the preceding closed-contact phase is considered negligible. The initial conditions of the arc current (*i*) and the internal resistance (*R*) during this phase are assumed to be equal to the final values of the previous phase, ensuring continuity of state variables common to both arc models.

The model described by (13) has nine state variables. A suitable choice for the state vector is

(14) $$x_{2}={\left[\begin{array}{ccccccccc} i & R & T_{P} & T_{A} & R_{P-A} & R_{A} & T_{E} & R_{P-E} & R_{E} \end{array}\right]}^{T}\;.$$

Similar to the model in the closed-contact phase, the dynamic behavior during the contact-opening phase can be described by rewriting (13) in the form of (3), incorporating the stochastic error term ($e_{2}\left(t\right)$), which quantifies the approximation provided by the model with the previously defined initial conditions:

(15) $$\frac{dx_{2}}{dt}=f_{2}\left(x_{2},v_{N}\right)+e_{2}\left(t\right).$$

Considering Equation (13) and the expression for the AACB voltage (see Figure 7a), i.e.,

(16) $$v=Ri+E+\left(L+i\frac{dL}{di}\right)\frac{di}{dt},$$

the measurands (4) can be expressed in terms of the state vector ($x_{2}$) and the driving voltage ($v_{N}$) through the function vector:

(17) $$h_{2}\left(x_{2},v_{N}\right)=\left[\begin{array}{c} x_{2}\left[1\right]\\ \frac{\left[L_{N}x_{2}\left[2\right]-\left(L+x_{2}\left[1\right]\frac{dL}{di}\right)R_{N}\right]x_{2}\left[1\right]+L_{N}E+\left(L+x_{2}\left[1\right]\frac{dL}{di}\right)v_{N}}{L+x_{2}\left[1\right]\frac{dL}{di}+L_{N}} \end{array}\right]$$

Here, square-bracket notation $x_{2}\left[m\right]$ denotes the *m*-th element of state vector $x_{2}$. To improve readability, explicit time dependencies are omitted in the equation.

### 3.3. Phase 3: Contacts Opened—Arc on Rails

The arc-on-rails model describes the process in which the arc moves away from the contacts and travels along the arc runners due to the forces acting upon it. As the arc progresses, it elongates due to the divergent shape of the rails, as illustrated in Figure 1.

**Figure 8:**
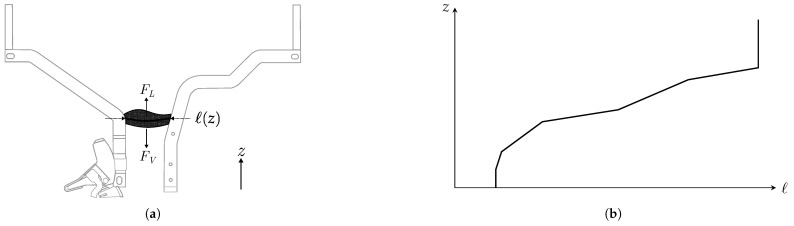
Mechanical model of the arc moving along the rails: (**a**) internal forces acting on the arc; (**b**) arc length l(z) as a function of the spatial coordinate (*z*).

Introducing the spatial coordinate (*z*), which specifies the arc plasma’s location along the rails (as shown in Figure 8a), the plasma length (l(z)) is a known function of *z*. An example of this relationship for a specific AACB is provided in Figure 8b.

The mechanical model governing the arc’s movement along the rails is derived from Newton’s second law:

(18) $$F_{L}-F_{V}=m\ddot{z}$$

where $F_{L}$ is the Lorentz force driving the arc, $F_{V}$ is the viscous air force opposing plasma movement and *m* represents the plasma’s mass. The Lorentz force is the primary factor contributing to the elongation of the arc column, significantly influencing the increase in arc voltage [16,17]. The effect of pressure differences is not explicitly considered, as it is included within the Lorentz force.

The Lorentz force is proportional to the product of the magnetic field, the electric arc current (i(t)) and the arc’s length (l(z)). Since the magnetic field itself is proportional to the plasma current, the Lorentz force can be expressed as

$$F_{L}\;\propto \;l\left(z\right)\;\cdot \;i{\left(t\right)}^{2}.$$

The viscous force follows the typical aerodynamic drag-force model [18], making it proportional to the square of the arc’s plasma velocity:

$$F_{V}\;\propto \;{\dot{z}}^{2}$$

Neglecting the plasma inertia in (18) (i.e., considering m≈0), a simplified mechanical model describing the arc movement along the rails is derived as

$$\dot{z}=k_{M}\;\cdot \;\sqrt{l(z)}\;\cdot \;i\left(t\right)$$

where $k_{M}$ is an appropriate proportionality constant.

**Figure 9:**
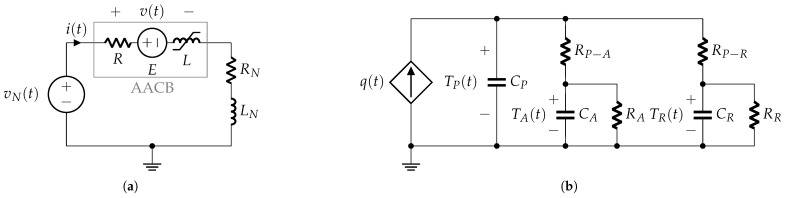
Model of the arc moving along the rails: (**a**) electrical equivalent circuit; (**b**) thermal equivalent circuit.

The electrical model shown in Figure 9a, although appearing similar to that of the previous phase, differs in the physical modeling of its parameters and their relationships with the new state variables of this phase model.

The internal inductance (*L*) is now primarily influenced by the position of the arc plasma. This dependence arises from two key factors: the increasing loop area associated with the magnetic flux lines as the arc coordinate (*z*) increases and the effect of the ferromagnetic material of the splitter plates. The inductance function (L(i,z)) is determined using finite-element method (FEM) simulations of the AACB. The dependence of inductance on the arc current (*i*) results from the magnetic saturation of the splitter plate material, whereas the iron core of the overcurrent tripping device (Section 2.1) remains fully saturated during this phase.

The internal resistance (*R*) is mainly determined by the resistance of the arc plasma. This resistance depends on both the arc length (*ℓ*) [19,20] and the plasma temperature ($T_{P}$) [21].

(19) $$R=R\left(l,T_{P}\right)=k_{R}\frac{l}{T_{P}^{1.5}}\;.$$

The thermal–electrical network shown in Figure 9b represents the lumped-parameter thermal model of the AACB when the arc is moving along the rails. This model is similar to the one discussed for the previous phase, with the difference being that heat transfer is now considered between the plasma and the rails instead of between the plasma and the electrodes. In this representation, the thermal resistance ($R_{P-R}$) (shown in Figure 9b) characterizes thermal conduction between the plasma and the rails, while the thermal capacity ($C_{R}$) denotes the heat capacity of the rails. Additionally, the thermal resistance ($R_{R}$) represents the heat conduction between the rail and the external environment beyond the arc chamber.

Assume that the following data are available, along with their associated uncertainties:

- The values of the external resistance ($R_{N}$) and inductance ($L_{N}$), as well as the supplying voltage ($v_{N}\left(t\right)$) (as in any phase model);
- The inductance (L(i,z)) and its derivatives (∂L/∂i and ∂L/∂z);
- The electromotive force (*E*) and the arc-length mapping (l(z));
- The thermal capacities of plasma, air and rails ($C_{P},\;C_{A}$ and $C_{R}$, respectively).

Also assume that the plasma resistance coefficient ($k_{R}$) and the thermal resistances ($R_{P-A}$ and $R_{P-R}$) are modeled as unknown, approximately constant quantities. Therefore, the equations governing the system are

(20) $$\left\{\begin{array}{cc} v_{N}=E+\left(R+R_{N}\right)i+\left(L+i\frac{\partial L}{\partial i}+L_{N}\right)\frac{di}{dt}+i\frac{\partial L}{\partial z}\frac{dz}{dt}\;, & i\left(t_{3}\right)=i\left(t_{3}^{-}\right),\;z\left(t_{3}\right)=\bar{z}\\ \frac{dk_{R}}{dt}=0\;, & k_{R}\left(t_{3}\right)=\frac{R\left(t_{3}^{-}\right)T_{P}{\left(t_{3}^{-}\right)}^{1.5}}{d}\\ \frac{dz}{dt}=k_{M}\;\cdot \;\sqrt{l(z)}\;\cdot \;i, & z\left(t_{3}\right)=\bar{z}\\ Ri^{2}+Ei=C_{P}\frac{dT_{P}}{dt}+C_{A}\frac{dT_{A}}{dt}+\frac{T_{A}}{R_{A}}+C_{R}\frac{dT_{R}}{dt}+\frac{T_{R}}{R_{R}}\;, & T_{P}\left(t_{3}\right)=T_{P}\left(t_{3}^{-}\right),\;T_{A}\left(t_{3}\right)=T_{A}\left(t_{3}^{-}\right),T_{R}\left(t_{3}\right)=T_{E}\left(t_{3}^{-}\right)\\ C_{A}\frac{dT_{A}}{dt}+\frac{T_{A}}{R_{A}}=\frac{T_{P}-T_{A}}{R_{P-A}}\;, & T_{A}\left(t_{3}\right)=T_{A}\left(t_{3}^{-}\right)\\ C_{R}\frac{dT_{R}}{dt}+\frac{T_{R}}{R_{R}}=\frac{T_{P}-T_{R}}{R_{P-R}}\;, & T_{R}\left(t_{3}\right)=T_{E}\left(t_{3}^{-}\right)\\ \frac{dR_{P-A}}{dt}=0\;, & R_{P-A}\left(t_{3}\right)=R_{P-A}\left(t_{3}^{-}\right)\\ \frac{dR_{A}}{dt}=0\;, & R_{A}\left(t_{3}\right)=R_{A}\left(t_{3}^{-}\right)\\ \frac{dR_{P-R}}{dt}=0\;, & R_{P-R}\left(t_{3}\right)=R_{P-E}\left(t_{3}^{-}\right)\\ \frac{dR_{R}}{dt}=0\;, & R_{R}\left(t_{3}\right)={\bar{R}}_{R}\\ \frac{dk_{M}}{dt}=0\;, & k_{M}\left(t_{3}\right)={\bar{k}}_{M} \end{array}\right.\;.$$

where time instant $t_{3}$ represents the initial time in the phase 3 model. The initial conditions include guessed values for the mechanical coefficient ($k_{M}$) and the thermal resistances of the rails ($R_{P-R}$ and $R_{R}$). The initial condition for $k_{R}$ is derived from Equation (19), where the arc length (*ℓ*) is replaced by the known contact separation distance (*d*) when the contacts are fully open. The initial arc position is set to the starting *z* coordinate of the rail, denoted as $\bar{z}$. The initial values of the arc current, arc temperature rise and air temperature rise are assumed to match the final values from the previous phase, ensuring continuity of these state variables during model phase switching. Additionally, the initial temperature rise of the rails is set to the electrode temperature rise at the end of the previous model phase.

The model described by (20) has eleven state variables. A suitable choice for the state vector is

(21) $$x_{3}={\left[\begin{array}{ccccccccccc} i & k_{R} & T_{P} & T_{A} & R_{P-A} & R_{A} & z & k_{M} & T_{R} & R_{P-R} & R_{R} \end{array}\right]}^{T}\;.$$

Similar to the model in the previous phases, the dynamic behavior during the arc-on-rails phase can be expressed by rewriting (20) in the form of (3), incorporating the stochastic error term ($e_{3}\left(t\right)$), which quantifies the approximation provided by the model with the previously defined initial conditions:

(22) $$\frac{dx_{3}}{dt}=f_{3}\left(x_{3},v_{N}\right)+e_{3}\left(t\right)$$

Considering Equation (20) and the expression for the AACB voltage (see Figure 9a), i.e.,

(23) $$v=Ri+E+\left(L+i\frac{\partial L}{\partial i}\right)\frac{di}{dt}+i\frac{\partial L}{\partial z}\frac{dz}{dt},$$

the measurands (4) can be expressed in terms of the state vector ($x_{3}$) and the driving voltage ($v_{N}$) through the function vector:

(24) $$h_{3}\left(x_{3},v_{N}\right)=\left[\begin{array}{c} x_{3}\left[1\right]\\ \frac{L_{N}\left(\frac{x_{3}\left[2\right]l\left(x_{3}\left[7\right]\right)}{x_{3}{\left[3\right]}^{1.5}}+x_{3}\left[1\right]\frac{\partial L}{\partial z}x_{3}\left[8\right]\sqrt{l\left(x_{3}\left[7\right]\right)}\right)x_{3}\left[1\right]+L_{N}E+\left(L+x_{3}\left[1\right]\frac{\partial L}{\partial i}\right)\left(v_{N}-R_{N}x_{3}\left[1\right]\right)}{L+x_{3}\left[1\right]\frac{\partial L}{\partial i}+L_{N}} \end{array}\right]\;.$$

Here, square-bracket notation $x_{3}\left[m\right]$ denotes the *m*-th element of state vector $x_{3}$. To improve readability, explicit time dependencies are omitted in the equation.

### 3.4. Phase 4: Contacts Opened—Arc Inside Splitter Plates

The arc-inside-the-splitter-plates model describes the process by which the arc interacts with some or all of the splitter plates. By dividing the arc, the splitter plates increase the number of arc roots, which, in turn, raises the total voltage drop due to the series of constant voltage drops at each arc root.

During this phase, the arc movement is highly complex, involving the rails and a varying number of splitter plates. To simplify the analysis, the plasma is assumed to remain fixed at a constant length equal to the total length of the splitter plates, eliminating the need for a mechanical model.

**Figure 10:**
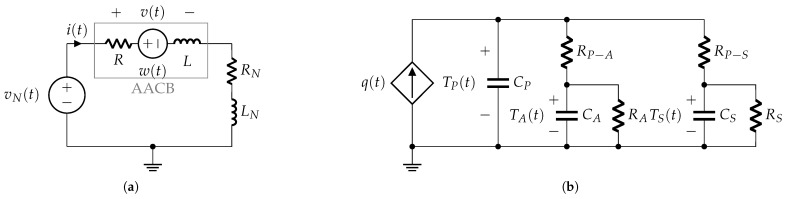
Model of the arc inside the splitter plates: (**a**) electrical equivalent circuit; (**b**) thermal equivalent circuit.

The electrical model shown in Figure 10a differs from that of the previous phase with the introduction of the internal electromotive force (w(t)). This term represents the sum of the voltage drops at the arc roots, which may vary over time depending on the number of splitter plates affected by the electrical arc.

The thermal–electrical network shown in Figure 10b represents the lumped-parameter thermal model of the AACB when the arc is inside the splitter plates. This model is similar to the one discussed for the previous phase, with the difference being that heat transfer is now considered between the plasma and the splitter plates instead of between the plasma and the rails. In this representation, the thermal resistance ($R_{P-S}$) (shown in Figure 10b) characterizes thermal conduction between the plasma and the splitter plates, while the thermal capacity ($C_{S}$) denotes the heat capacity of the splitter plates. Additionally, the thermal resistance ($R_{S}$) represents the heat conduction between the splitter plates and the external environment beyond the arc chamber.

Assume that the following data are available, along with their associated uncertainties:

- The values of the external resistance ($R_{N}$) and inductance ($L_{N}$), as well as the supplying voltage ($v_{N}\left(t\right)$);
- The inductance (*L*) when the arc is inside the splitter plates;
- The thermal capacities of plasma, air and splitter plates ($C_{P},\;C_{A}$ and $C_{S}$, respectively);

Also assume that the plasma resistance coefficient ($k_{R}$) and the thermal resistances ($R_{P-A}$ and $R_{P-S}$) remain constant. Therefore, the equations governing the system are

(25) $$\left\{\begin{array}{cc} v_{N}=w+\left(R+R_{N}\right)i+\left(L+L_{N}\right)\frac{di}{dt}\;, & i\left(t_{4}\right)=i\left(t_{4}^{-}\right)\\ \frac{dk_{R}}{dt}=0\;, & k_{R}\left(t_{4}\right)=k_{R}\left(t_{4}^{-}\right)\\ \frac{dw}{dt}=0\;, & w(t_{4})=E\\ Ri^{2}+Ei=C_{P}\frac{dT_{P}}{dt}+C_{A}\frac{dT_{A}}{dt}+\frac{T_{A}}{R_{A}}+C_{S}\frac{dT_{S}}{dt}+\frac{T_{S}}{R_{S}}\;, & T_{P}\left(t_{4}\right)=T_{P}\left(t_{4}^{-}\right),\;T_{A}\left(t_{4}\right)=T_{A}\left(t_{4}^{-}\right),T_{S}\left(t_{4}\right)=T_{R}\left(t_{4}^{-}\right)\\ C_{A}\frac{dT_{A}}{dt}+\frac{T_{A}}{R_{A}}=\frac{T_{P}-T_{A}}{R_{P-A}}\;, & T_{A}\left(t_{4}\right)=T_{A}\left(t_{4}^{-}\right)\\ C_{S}\frac{dT_{S}}{dt}+\frac{T_{S}}{R_{S}}=\frac{T_{P}-T_{S}}{R_{P-S}}\;, & T_{S}\left(t_{4}\right)=T_{R}\left(t_{4}^{-}\right)\\ \frac{dR_{P-A}}{dt}=0\;, & R_{P-A}\left(t_{4}\right)=R_{P-A}\left(t_{4}^{-}\right)\\ \frac{dR_{A}}{dt}=0\;, & R_{A}\left(t_{4}\right)=R_{A}\left(t_{4}^{-}\right)\\ \frac{dR_{P-S}}{dt}=0\;, & R_{P-S}\left(t_{4}\right)={\bar{R}}_{P-S}\\ \frac{dR_{S}}{dt}=0\;, & R_{S}\left(t_{4}\right)={\bar{R}}_{S} \end{array}\right.\;.$$

At time instant $t_{4}$, which marks the beginning of the phase 4 model, the initial conditions include estimated values for the thermal resistances of the splitter plates ($R_{P-S}$ and $R_{S}$). Additionally, the initial total voltage drop at the arc roots (*w*) is set equal to the voltage drop at the arc roots (*E*) from the previous phase.

The initial values of the arc current, arc temperature rise and air temperature rise are carried over from the final values of the previous phase, ensuring continuity of these state variables during the transition between model phases. Furthermore, the initial temperature rise of the splitter plates is set to match the rail temperature rise at the end of the preceding model phase.

The model described by (25) has ten state variables. A suitable choice for the state vector is

(26) $$x_{4}={\left[\begin{array}{cccccccccc} i & k_{R} & T_{P} & T_{A} & R_{P-A} & R_{A} & T_{S} & R_{P-S} & R_{S} & w \end{array}\right]}^{T}\;.$$

Similar to the model in the previous phases, the dynamic behavior during the arc-on-rails phase can be expressed by rewriting (25) as with the previously defined initial conditions:

(27) $$\frac{dx_{4}}{dt}=f_{4}\left(x_{4},v_{N}\right)+e_{4}\left(t\right)$$

The stochastic error term ($e_{4}\left(t\right)$) quantifies the approximation provided by the model.

Considering Equation (25) and the expression for the AACB voltage (see Figure 10a), i.e.,

(28) $$v=Ri+w+L\frac{di}{dt},$$

the measurands (4) can be expressed in terms of the state vector ($x_{4}$) and the driving voltage ($v_{N}$) through the function vector:

(29) $$h_{4}\left(x_{4},\;v_{N}\right)=\left[\begin{array}{c} x_{4}\left[1\right]\\ \frac{L_{N}\left(x_{4}\left[10\right]+\frac{x_{4}\left[2\right]\bar{l}}{x_{4}{\left[3\right]}^{1.5}}x_{4}\left[1\right]\right)-LR_{N}x_{4}\left[1\right]}{L+L_{N}}+\frac{L}{L+L_{N}}v_{N} \end{array}\right]\;.$$

Here, square-bracket notation $x_{4}\left[m\right]$ denotes the *m*-th element of state vector $x_{4}$. To improve readability, explicit time dependencies are omitted in the equation.

## 4. Extended Kalman Filter for State Estimation

In data assimilation and optimal estimation, the Kalman filter is a widely used approach [22,23,24] consisting of two main steps.

In the first step, the mean and variance of the probability density function (PDF) representing the forecast of the state variables at desired time instants are obtained through prediction using the given dynamical models ($f_{j}\left(\;\cdot \;\right)$, where j=1,…,4).

In the second step, once measurements have been collected, the predicted state variables are corrected based on the mismatch between the observed data and the predicted values derived using the given model ($h_{j}\left(\;\cdot \;\right)$, where j=1,…,4) that relates the measured data to the state variables. This correction is applied linearly, and the correction matrix, known as the Kalman gain matrix, is computed to ensure unbiased estimators while minimizing the sum of the variances of the final estimators [22,23].

Since the final estimate is obtained by adjusting model-based predictions using discrepancies with measured data, this procedure is referred to as data assimilation.

The Kalman filter exhibits optimal properties under the assumption that the error PDFs are normally distributed and that both the process model and the measurement model are linear. In cases where these models exhibit nonlinearity, the relationships are linearized, leading to an alternative estimation algorithm known as the extended Kalman filter [22,23].

With reference to the discrete-time models defined in (5) and the associated error covariance matrices in (6), the prediction step of the Kalman filter is defined by the following equations:

(30) $$\left\{\begin{array}{c} x_{j}^{F}\left(k\right)=x_{j}^{A}\left(k-1\right)+\Delta t_{S}\;\cdot \;f_{j}\left(x_{j}^{A}\left(k-1\right),v_{N}\left(k-1\right)\right)\\ P_{j}^{F}\left(k\right)=F_{j}\left(k-1\right)P_{j}^{A}\left(k-1\right)F_{j}{\left(k-1\right)}^{T}+Q_{j}\left(k-1\right) \end{array}\right.$$

where the state transition matrix ($F_{j}\left(k-1\right)$) is expressed as the following Jacobian matrix:

(31) $$F_{j}\left(k-1\right)=\Delta t_{S}{\left.\frac{\partial f_{j}}{\partial x_{j}^{T}}\right|}_{x_{j}=x_{j}^{A}\left(k-1\right)}$$

Here, $x_{j}^{F}\left(k\right)$ represents the predicted (forecasted) state vector at discrete time step *k*, while $P_{j}^{F}\left(k\right)$ denotes the covariance matrix of the predicted state estimate. The state transition matrix ($F_{j}\left(k-1\right)$) is obtained by linearizing the system dynamics around the most recently assimilated state ($x_{j}^{A}\left(k-1\right)$), whose uncertainty is characterized by the covariance matrix ($P_{j}^{A}\left(k-1\right)$).

The assimilation step is performed according to the following equations:

(32) $$\left\{\begin{array}{c} x_{j}^{A}\left(k\right)=x_{j}^{F}\left(k\right)+K_{j}\left(k\right)\left[y\left(k\right)-h_{j}\left(x_{j}^{F}\left(k\right),v_{N}\left(k\right)\right)\right]\\ P_{j}^{A}\left(k\right)=\left(I-K_{j}\left(k\right)H_{j}\left(k\right)\right)P_{j}^{F}\left(k\right){\left(I-K_{j}\left(k\right)H_{j}\left(k\right)\right)}^{T}+K_{j}\left(k\right)R\left(k\right)K_{j}{\left(k\right)}^{T} \end{array}\right.$$

where the Jacobian matrix ($H_{j}\left(k\right)$) is expressed as

(33) $$H_{j}\left(k\right)={\left.\frac{\partial h_{j}}{\partial x_{j}^{T}}\right|}_{x_{j}=x_{j}^{F}\left(k\right)}$$

Here, $x_{j}^{A}\left(k\right)$ represents the updated (assimilated) state vector at discrete time step *k*, incorporating the measurement information. The updated covariance matrix ($P_{j}^{A}\left(k\right)$) accounts for both the forecast and the measurement errors, ensuring an optimal balance between model predictions and observed data.

The Kalman gain matrix ($K_{j}\left(k\right)$) optimally updates the forecasted state ($x_{j}^{F}\left(k\right)$) based on the discrepancy between the observed data (y(k)) and the model-predicted observations ($h_{j}\left(x_{j}^{F}\left(k\right),v_{N}\left(k\right)\right)$). The gain matrix is computed as

(34) $$K_{j}\left(k\right)=P_{j}^{F}\left(k\right)H_{j}{\left(k\right)}^{T}{\left[H_{j}\left(k\right)P_{j}^{F}\left(k\right)H_{j}{\left(k\right)}^{T}+R\left(k\right)\right]}^{-1}$$

## 5. Experimental Results

As already mentioned in Section 2.1, a DC AACB prototype was used to perform dedicated short-circuit tests according to the IEC 61992-2 standard (duty “f” test cycle) [10] as most the representative and severe scenario of maximum fault current. From this perspective, the presented model could be beneficial in terms of providing a preliminary evaluation of the interruptive performance of AACBs, reducing the number of expensive and destructive tests during product development.

**Figure 11:**
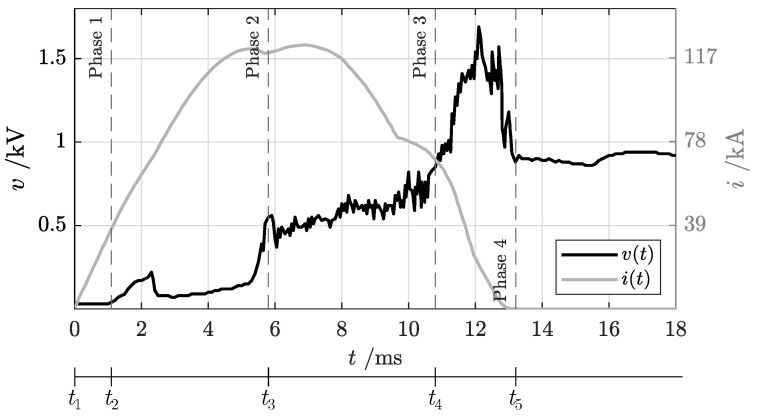
Voltage and current waveforms measured at the AACB terminals during the test and used as input signals for the extended Kalman filter implementation. The figure also highlights the four arc phases manually identified to correspond with the four arc models employed within the Kalman filter framework.

In Figure 11, a typical duty “f” shot is presented with the following test parameters: supply voltage of $v_{N}\;=\;920\;V$, prospective sustained short-circuit current of $I_{ss}\;=\;125\;\mathrm{kA}$ with a peak value of ${\hat{I}}_{ss}\;=\;178\;\mathrm{kA}$ and an initial current rise rate of di/dt = 37.7 kA/ms. The overcurrent trip setting was configured at 12 kA.

The test circuit implemented during the experiment follows the IEC 61992-1 standard [25], and tests were conducted under controlled environmental conditions: an ambient temperature of 21 °C, atmospheric pressure of 1008 hPa and relative humidity of 55%. The test network is illustrated in Figure 3a and consists of the following components:

- AC side: A 50 Hz, 10.2 kV three-phase generator supplies power to a 10.2/0.65 kV Yy transformer through a 0.2 Ω resistance and 3 mH inductance line.
- DC side: An uncontrolled full-bridge rectifier feeds the test object via rectangular busbars, which introduce unintentional parasitic impedance.
- Test object: The tested device is a prototype DC circuit breaker. It has a nominal voltage of 900 V, a 2.6 kA service current and a 178/125 kA rated short-circuit and breaking capacity.

The measurement system used during testing is characterized as follows:

- Current sensor: A coaxial shunt with an 18.79 kA/V scale factor, 10 kHz bandwidth and maximum current rating of 200 kA for a 100 ms duration. The shunt was calibrated in accordance with IEC 62475 standard [26].
- Voltage sensor: An RC voltage divider rated at 6.3 kV, with an input impedance of 20 MΩ and 25 pF and a bandwidth of 300 kHz. Calibration was performed following IEC 60060-2:2025 standard [27].

### 5.1. Identification of Short-Circuit Oscillogram Phases

The voltage–current oscillogram represents a first quantitative output from the short-circuit test that allows for examination of fault breaking capacity. Usually, oscillogram analysis is performed by expert operators with deep knowledge of AACB details and arc phenomena. Some peculiarities of voltage–current signals help to extract additional information about arc evolution, such as the arc’s position and its stability in the AACB. In the oscillogram shown in Figure 11, time intervals related to the different phases listed in Section 3 can be identified. The dotted lines represent the ending points the phases.

- Phase 1 is characterized by zero voltage across the AACB terminals and an increasing current flow in the breaker conductive path, following the prospective current profile. During this phase, the contacts are closed with the nominal contact pressure. When the current value overcomes the threshold trip level set for the overcurrent device, the tripping mechanism starts to react to short-circuit fault and to drive contact opening. This action requires some time, typically in the order of a maximum of a few ms for an high-speed ACCB, during which time the contacts remain in closed position.
- Phase 2: The transition to this phase can be recognized by a constant build-up in the voltage level. During this phase, contacts are opening, and the distance between moving and fixed contacts increases. An electric arc starts to form and to gradually elongate, leading the voltage drop across the ACCB to progressively increase. The voltage rise rate depends on the final opening velocity of the ACCB mechanism as a result of the opening force provided by the mechanical actuators and the electro-dynamic repulsive forces generated by the fault current. The first part of this phase (between approximately 1 and 5 ms) is characterized by a lower slope of voltage increase because the arc is still rooted on the main contacts and the arc length is limited to the contact stroke. The last part of this stage presents an abrupt change in current rise, corresponding to arc commutation from contacts to arc rails, which is the transition point to the following stage.
- Phase 3: During this phase, the arc remains on arc rails. Their duty is to further elongate the electric arc and to guide it towards the arc chute. The duration of this phase strongly depends on the AACB geometry—in particular, the geometries of the contacts and rails. The AACB breaking capability is already visible at this stage: the current rise is limited, and therefore, its gradient starts to decrease, deviating from the peak prospective current profile. Due to the complex balance between electro-dynamic, magnetic and aerodynamic forces, the arc is increasingly stretched along the rails, but this process is highly unstable as can be observed from the numerous fluctuations in voltage signal. During this stage, it is critical to support the continuous “build-up” of a counter voltage in the ACCB and “push” the current towards the zero crossing and successful current interruption. The duration of this phase strongly contributes to total arc break time.
- Phase 4: Once the electric arc definitely enters the arc chute, a more rapid voltage increase can be observed until the maximum voltage level is reached. In this phase, the splitter plates act to divide the original energetic arc into smaller arcs. It is crucial that the rails and arc chute are properly designed to avoid any arc re-ignition towards the contact region, which would result in an evident voltage drop to levels close to those of phase 1 and phase 2, an increase in the real fault current and a severe increase in total breaking time. Once the fault is permanently interrupted, the current goes to zero and the supply voltage level is fully restored.

### 5.2. Measurement and Model Uncertainties

The measurement and model uncertainties are specified by covariance matrices $R_{j}\left(k\right)$ and $Q_{j}\left(k\right)$, respectively, corresponding to the stochastic processes ($\epsilon_{j}\left(k\Delta t_{S}\right)$ and $\Delta t_{S}\;\cdot \;e_{j}\left(k\Delta t_{s}\right)$) in Equation (6).

The measurement error covariance matrix ($R_{j}\left(k\right)$) is computed by considering that the measurement relationship in (5) can be rewritten for each phase as

(35) $$y\left(k\right)=h_{j}\left(x_{j}\left(k\right),\;v_{N}\left(k\right),\;\pi_{j}\left(k\right)\right)+\epsilon^{\prime }\left(k\right)$$

to account for its dependence on specific parameters organized in $\pi_{j}\left(k\right)$. These parameters, along with the supplied voltage ($v_{N}\left(k\right)$), are subject to errors ($e_{\pi_{j}}\left(k\right)$ and $e_{v_{N}}\left(k\right)$, respectively). Here, $\epsilon^{\prime }\left(k\right)$ represents the measurement error in the current and voltage introduced solely by the measuring instruments.

Using a first-order Taylor expansion, the total error ($\epsilon_{j}$) affecting the data can be approximated as

(36) $$\epsilon_{j}\approx \epsilon^{\prime }+\frac{\partial h_{j}}{\partial v_{N}}e_{v_{N}}+\frac{\partial h_{j}}{\partial \pi_{j}^{T}}e_{\pi_{j}}$$

To improve readability, the dependence on discrete time *k* is omitted. Assuming that errors $\epsilon^{\prime },\;e_{v_{N}}$ and $e_{\pi_{j}}$ are mutually uncorrelated, the associated covariance matrix can be expressed as

(37) $$R_{j}\left(k\right)\approx R_{\epsilon^{\prime }}+\sigma_{v_{N}}^{2}\frac{\partial h_{j}}{\partial v_{N}}\frac{\partial h_{j}^{T}}{\partial v_{N}}+\frac{\partial h_{j}}{\partial \pi_{j}^{T}}R_{\pi_{j}}\frac{\partial h_{j}^{T}}{\partial \pi_{j}}$$

Hereafter, $\sigma_{a}^{2}$ and $R_{a}$ represent the variance of the error affecting the scalar quantity (*a*) and the covariance matrix of the errors affecting the quantities of vector a, respectively.

The covariance matrix ($R_{\epsilon^{\prime }}$) is identical for all models, as the measured data are acquired from the same instruments. These instruments sample the instantaneous values of the current (i(t)) and voltage (v(t)) with a sampling period of $\Delta t_{S}=10\;\mu s$. Moreover, $R_{\epsilon^{\prime }}$ is a diagonal matrix, as measurement errors are mutually uncorrelated.

Furthermore, the covariance matrix ($R_{\epsilon^{\prime }}$) is considered stationary, since the standard deviations of the measurement errors are also assumed to be constant, with values of $\sigma_{i}\;=\;2\;\mathrm{kA}\;$ and $\sigma_{v}\;=\;0.02\;\mathrm{kV}$ for the current and voltage measurement errors, respectively, as declared in the official test report from the laboratory. Consequently, the covariance matrix of the error vector ($\epsilon^{\prime }$) is expressed as

(38) $$R_{\epsilon^{\prime }}=\left[\begin{array}{cc} \sigma_{i}^{2} & 0\\ 0 & \sigma_{v}^{2} \end{array}\right]=\left[\begin{array}{cc} 4\times 10^{6}\;A^{2}\; & 0\\ 0 & 4\times 10^{2}\;V^{2} \end{array}\right]$$

The measured current and voltage range from 0 kA to 123 kA and from 0.02 kV to 1.69 kV, respectively. Each model phase has its own stochastic error vector ($e_{\pi_{j}}$), with a corresponding covariance matrix. While each model phase has its own distinct parameters, some terms contribute to all model phases. These common parameters are associated with the laboratory setup, namely the resistance ($R_{N}$) and inductance ($L_{N}$), as well as the supply voltage ($v_{N}\left(t\right)=V_{N}$), which is assumed to be constant. The values of these parameters are provided in Table 1. Hereafter, the values in parentheses denote the standard deviations, which are expressed in terms of the least significant digit of the corresponding parameter value [28,29].

The model error covariance matrix $Q_{j}\left(k\right)$ is computed similarly, considering that the model relationship in (5) for each phase can be rewritten as

(39) $$x_{j}\left(k\right)=x_{j}\left(k-1\right)+\Delta t_{S}\;\cdot \;f_{j}\left(x_{j}\left(k-1\right),v_{N}\left(k-1\right),\xi_{j}\left(k-1\right)\right)+\Delta t_{S}\;\cdot \;e_{j}^{\prime }\left(k-1\right),$$

accounting for its dependence on specific parameters organized in $\xi_{j}$. These parameters, along with the voltage ($v_{N}$), are subject to errors of $e_{\xi_{j}}$ and $e_{v_{N}}$, respectively. Here, $e_{j}^{\prime }$ represents the error vector introduced due to the simplified *j*-th model assumptions.

Using a first-order Taylor expansion, the total error ($e_{j}$) affecting the model can be approximated as

(40) $$e_{j}\approx e_{j}^{\prime }+\frac{\partial f_{j}}{\partial v_{N}}e_{v_{N}}+\frac{\partial f_{j}}{\partial \xi_{j}^{T}}e_{\xi_{j}}$$

Assuming that errors $e_{j}^{\prime },\;e_{v_{N}}$ and $e_{\xi_{j}}$ are mutually uncorrelated, the associated covariance matrix can be expressed as

(41) $$Q_{j}\left(k\right)\approx Q_{e_{j}^{\prime }}+\sigma_{v_{N}}^{2}\frac{\partial f_{j}}{\partial v_{N}}\frac{\partial f_{j}^{T}}{\partial v_{N}}+\frac{\partial f_{j}}{\partial \xi_{j}^{T}}Q_{\xi }\frac{\partial f_{j}^{T}}{\partial \xi_{j}}.$$

The error sensitivity coefficients in (37) and (41) were analyzed to identify the most significant contributions to the measurement and model covariance matrices. The results of this analysis for each model phase are presented and discussed in the following sections, along with the initial values of the assimilated state vector ($x_{j}^{A}\left(k_{j}\right)$) and its corresponding covariance matrix ($P_{j}^{A}\left(k_{j}\right)$, where $k_{j}$ represents the initial discrete time of each *j*-th model phase).

#### 5.2.1. Phase 1: Contacts Closed

The parameters influencing the measurement and model uncertainties in this phase are represented by

(42) $$\pi_{1}=\xi_{1}={\left[\begin{array}{ccccc} v_{N} & L_{N} & R_{N} & L & dL/di \end{array}\right]}^{T}$$

The values and standard deviations of $v_{N},\;L_{N}$ and $R_{N}$ are provided in Table 1. The standard deviations of the error contributions due to the internal inductance (*L*) and its derivative were assumed to be 20% of their respective values. The inductance is a function of current *i*, derived using a FEM model for the AACB overcurrent trip device, and its derivatives are approximated by

(43) $$\frac{L(i)}{\mu H}=3.1{\left(\frac{\left|i\right|}{\mathrm{kA}}\right)}^{-0.48},\;\frac{dL/\mu H}{di/\mathrm{kA}}=-1.5\frac{i}{\left|i\right|}{\left(\frac{\left|i\right|}{\mathrm{kA}}\right)}^{-1.48}$$

Considering the error sensitivity coefficients in the covariance propagation law (37) for this model phase, the main contribution to the measurement model error covariance matrix is expressed by (38). Consequently, the measurement error covariance matrix is expressed as

(44) $$R_{1}\left(k\right)=R_{\epsilon^{\prime }}=\left[\begin{array}{cc} 4\times 10^{6}\;A^{2} & 0\\ 0 & 4\times 10^{2}\;V^{2} \end{array}\right]$$

Since this matrix remains constant over time, the measurement error covariance is considered stationary.

The error sensitivity coefficients in (41) indicate that the primary contribution to the error model of the first state variable (the current) arises from errors in the external network voltage ($v_{N}$) and inductance ($L_{N}$). This results in a standard deviation of 20 A.

The error model for the second state variable (the contact resistance), on the other hand, is independent of any parameters. In this case, the error in the model equation solely accounts for the approximation inherent in the model. Since the contact resistance, which is modeled as a constant, is expected to increase at a rate of approximately 90 μΩ/m$s^{-1}$ during this phase and given the sampling period of $\Delta t_{S}\;=\;10\;\mu s$, the model error is assumed to be uniformly distributed within the range of −0.9 μΩ to 0.9 μΩ. This assumption results in a standard deviation of 0.5 μΩ.

Consequently, the model error covariance matrix is expressed as

(45) $$Q_{1}\left(k\right)=\left[\begin{array}{cc} 4\times 10^{2}\;A^{2} & 0\\ 0 & 3\times 10^{-13}\;\Omega^{2} \end{array}\right]$$

Since this matrix remains constant over time, the model error covariance is considered stationary.

The initialization of the assimilated state variables and their covariance matrix is empirically defined as follows:

(46) $$x_{1}^{A}\left(k_{1}\right)=\left[\begin{array}{c} 0.0\;kA\\ 10\;\mu \Omega \end{array}\right],\;P_{1}^{A}\left(k_{1}\right)=\left[\begin{array}{cc} 1\times 10^{4}\;A^{2} & 0\\ 0 & 9\times 10^{-12}\;\Omega^{2} \end{array}\right]$$

where $k_{1}=t_{1}/\Delta t_{S}$ denotes the initial discrete time of the first model phase.

#### 5.2.2. Phase 2: Contact Opening

During this phase, the nonlinear inductance of the tripping mechanism in the analyzed device becomes saturated; thus, the dL/di parameter is eliminated. The parameters that influence measurement and model uncertainties in this phase are expressed as

(47) $$\pi_{2}={\left[\begin{array}{ccccc} v_{N} & L_{N} & R_{N} & L & E \end{array}\right]}^{T}\;\mathrm{and}\;\xi_{2}={\left[\begin{array}{cccccccc} v_{N} & L_{N} & R_{N} & L & E & C_{P} & C_{A} & C_{E} \end{array}\right]}^{T}$$

The values and standard deviations of $v_{N},\;L_{N}$ and $R_{N}$ are provided in Table 1. The value and standard deviation of the error contributions due to the internal inductance (*L*) follow the same expression as described in the previous section.

The values and standard deviations of the parameters for this phase, except for those related to the laboratory setup presented in Table 1, are provided in Table 2.

Considering the error sensitivity coefficients in the covariance propagation law (37) for this model phase, the primary contribution to the measurement model error covariance matrix, apart from (38), arises from the error affecting the supply voltage ($v_{N}$). However, this contribution remains negligible compared to the instrument-related contributions in (38). As a result, the measurement error covariance matrix is expressed as

(48) $$R_{2}\left(k\right)=R_{\epsilon^{\prime }}=\left[\begin{array}{cc} 4\times 10^{6}\;A^{2} & 0\\ 0 & 4\times 10^{2}\;V^{2} \end{array}\right]$$

Since this matrix remains constant over time, the measurement error covariance is considered stationary.

For the analyzed AACB, the thermal time constant ($R_{A}C_{A}$), given typical values of $C_{A}$ and $R_{A}$, is approximately 50 s. Similarly, the time constant ($R_{E}C_{E}$) is around 450 s. Since both time constants are significantly larger than the duration of the contact-opening phase, thermal exchange with the external environment is considered adiabatic. Consequently, the thermal resistances ($R_{A}$ and $R_{E}$) in Figure 7b can be disregarded.

This simplification reduces the number of model equations in (13) and the state variables in (14) for this phase from nine to seven, as expressed by

(49) $$x_{2}={\left[\begin{array}{ccccccc} i & R & T_{P} & T_{A} & R_{P-A} & T_{E} & R_{P-E} \end{array}\right]}^{T}\;.$$

The error sensitivity coefficients in (41) indicate that the primary contributions to the error model stem from errors in the external network voltage ($v_{N}$), inductance ($L_{N}$) and arc-root voltage (*E*). Additionally, an a posteriori analysis confirmed that the error in the arc root voltage (*E*) is the dominant factor influencing the variance of the arc temperature error. Since this term is time-variant, the model covariance matrix is not stationary. Consequently, the model error covariance matrix is expressed as

(50) $$Q_{2}\left(k\right)=\mathrm{diag}\left\{\left[\begin{array}{c} 3\times 10^{2}\;A^{2}\\ 1\times 10^{-10}\;\Omega^{2}\\ \Delta t_{S}^{2}{\frac{\partial f_{2}\left[3\right]\left(k\right)}{\partial E}}^{2}\sigma_{E}^{2}\\ 1\;K^{2}\\ 2\times 10^{-13}\;K^{2}/W^{2}\\ 1\;K^{2}\\ 2\times 10^{-13}\;K^{2}/W^{2} \end{array}\right]\right\}$$

The initialization of the assimilated state variables and their covariance matrix, in accordance with (13), is performed using the values of the current (*i*) and the resistance (*R*) to ensure continuity with the previous phase.

All temperature rises are set to zero. The initial errors values were empirically assigned as uniformly distributed within the range of −50 K to 50 K for the arc temperature ($T_{P}$) and within the range of −15 K to 15 K for the air and electrode temperatures ($T_{A}$ and $T_{E}$, respectively).

The thermal resistances and their standard deviations were initially determined empirically using the values reported in (51) based on straightforward thermal analogies with finite-volume heat sinks and a sensitivity analysis of this analogy.

Consequently the initial values of the assimilated state variables and their covariance matrix are defined as follows

(51) $$x_{2}^{A}\left(k_{2}\right)=\left[\begin{array}{c} x_{1}^{A}\left[1\right]\left(k_{2}-1\right)\\ x_{1}^{A}\left[2\right]\left(k_{2}-1\right)\\ 0\;K\\ 0\;K\\ 10.0\times 10^{-4}\;K/W\\ 0\;K\\ 2.00\times 10^{-4}\;K/W \end{array}\right],\;P_{2}^{A}\left(k_{2}\right)=\mathrm{diag}\left\{\left[\begin{array}{c} P_{1}^{A}\left[1,1\right]\left(k_{2}-1\right)\\ P_{1}^{A}\left[2,2\right]\left(k_{2}-1\right)\\ 8\times 10^{2}\;K^{2}\\ 80\;K^{2}\\ 1\times 10^{-8}\;K^{2}/W^{2}\\ 80\;K^{2}\\ 5\times 10^{-10}K^{2}/W^{2} \end{array}\right]\right\}$$

where $k_{2}=t_{2}/\Delta t_{S}$ denotes the initial discrete time of the second model phase.

#### 5.2.3. Phase 3: Contacts Opened—Arc on Rails

During the third phase of the arc model, the internal inductance of the AACB depends on the arc’s position along the rails. Additionally, it is influenced by the current intensity due to significant ferromagnetic effects from the splitter plates. However, in the measurement model Equation (24), the resistance contribution, i.e.,

(52) $$\Delta R=i\frac{\partial L}{\partial z}k_{M}\sqrt{l},$$

is two orders of magnitude smaller than the arc resistance. Therefore, the error introduced by the nonlinearity (∂L/∂z) can be neglected in this model. The parameters affecting the measurement uncertainties in this phase are expressed as

(53) $$\pi_{3}={\left[\begin{array}{cccccc} v_{N} & L_{N} & R_{N} & L & \partial L/\partial i & E \end{array}\right]}^{T}$$

In the first differential equation in (20), the voltage term is bounded as follows:

(54) $$\left|\Delta V=i\frac{\partial L}{\partial z}\frac{dL}{dt}\right|\leq 8\;V.$$

Thus, the ∂L/∂z parameter is negligible in the arc model equations as well. Similarly, the term associated with inductance variation with respect to current, i.e.,

(55) $$\Delta L=i\frac{\partial L}{\partial i}\leq 100\;nH,$$

is also negligible in the first equation of (20). Therefore, the parameters influencing arc model uncertainties in this phase are expressed as

(56) $$\xi_{3}={\left[\begin{array}{cccccccc} v_{N} & L_{N} & R_{N} & L & E & C_{P} & C_{A} & C_{R} \end{array}\right]}^{T}$$

The values and standard deviations of $v_{N},\;L_{N}$ and $R_{N}$ are provided in Table 1. The values and standard deviations of the parameters for this phase, except for those related to the laboratory setup presented in in Table 1, are provided in Table 3.

Considering the error sensitivity coefficients in the covariance propagation law (37) for this model phase, the primary contribution to the measurement model error covariance matrix, apart from (38), arises from the error affecting the supply voltage ($v_{N}$). However, this contribution remains negligible compared to the instrument-related contributions in (38). As a result, the measurement error covariance matrix is expressed as

(57) $$R_{3}\left(k\right)=R_{\epsilon^{\prime }}=\left[\begin{array}{cc} 4\times 10^{6}\;A^{2} & 0\\ 0 & 4\times 10^{2}\;V^{2} \end{array}\right]$$

Since this matrix remains constant over time, the measurement error covariance is considered stationary.

For the analyzed AACB, the thermal time constant ($R_{R}C_{R}$) is significantly larger than the duration of the arc-on-rails phase, given typical values of $C_{R}$ and $R_{R}$. This is primarily due to the limited effective contact area between the rails and the external environment, which significantly restricts heat dissipation. As a result, the heat transfer process closely approximates an adiabatic condition, and thermal exchange with the external environment is considered negligible. Consequently, the thermal resistance ($R_{R}$) in Figure 9b can be disregarded.

However, the thermal resistance ($R_{A}$) cannot be neglected, as the turbulent heat exchange occurring in the arc chamber during this phase makes the time constant ($R_{A}C_{A}$) comparable to the duration of phase 3.

This simplification reduces the number of model equations in (20) and the state variables in (21) from eleven to ten, as expressed by

(58) $$x_{3}={\left[\begin{array}{cccccccccc} i & k_{R} & T_{P} & T_{A} & R_{P-A} & R_{A} & z & k_{M} & T_{R} & R_{P-R} \end{array}\right]}^{T}\;.$$

The error sensitivity coefficients in (41) indicate that the primary contributions to the error model stem from errors in the external network voltage ($v_{N}$), inductance ($L_{N}$) and arc length (*ℓ*). Additionally, an a posteriori analysis confirmed that the error in the arc length (*ℓ*) is the dominant factor influencing the variance of the arc temperature error. Since this term is time-variant, the model covariance matrix is not stationary. The error value for $k_{R}$ was empirically set, presuming variations would remain within a 10% margin during the phase. As a result, the standard deviation is defined as 4 ΩK^1.5^/m. Thermal resistances and their standard deviations are allocated as in the prior phase.

Consequently, the model error covariance matrix is expressed as

(59) $$Q_{3}\left(k\right)=\mathrm{diag}\left\{\left[\begin{array}{c} 2\times 10^{4}\;A^{2}\\ 20\;\Omega^{2}K^{2.25}/m^{2}\\ \Delta t_{S}^{2}{\left(\frac{\partial f_{3}\left[3\right]\left(k\right)}{\partial l}\right)}^{2}\sigma_{l}^{2}\\ 0\;K^{2}\\ 2\times 10^{-13}\;K^{2}/W^{2}\\ 2\times 10^{-13}\;K^{2}/W^{2}\\ 0\;m^{2}\\ 1\times 10^{-12}\;m^{4}/s^{2}A^{2}\\ 0\;K^{2}\\ 2\times 10^{-13}\;K^{2}/W^{2} \end{array}\right]\right\}$$

The initialization of the assimilated state variables and their covariance matrix, as defined in (20), is carried out using the current (*i*), the temperature rises ($T_{P},\;T_{A}$ and $T_{R}$), and the thermal resistances ($R_{P-A}$ and $R_{P-R}$) to ensure continuity with the previous phase. Specifically, continuity for the rail temperature rise is established by aligning it with the electrode temperature rise from the previous phase.

The thermal resistance ($R_{A}$) could not be defined with continuity from the previous state due to the removal of this state variable. Therefore, its value was empirically assigned as 0.50(2) mK/W, accounting for the effect of convective turbulent airflow in the arc chamber.

For the $k_{R}$ coefficient, according to (20), the initial condition is expressed as

(60) $$k_{R}\left(t_{3}\right)=\frac{R\left(t_{3}^{-}\right)T_{P}{\left(t_{3}^{-}\right)}^{1.5}}{d}$$

In the discrete-time formulation, the quantities of $R(t_{3}^{-})$ and $T_{P}\left(t_{3}^{-}\right)$ are assigned to the assimilated states from phase 2 according to the following relationships:

(61) $$R\left(t_{3}^{-}\right)\Rightarrow x_{2}^{A}\left[2\right]\left(k_{3}-1\right),\;\mathrm{and}\;T_{P}\left(t_{3}^{-}\right)\Rightarrow x_{2}^{A}\left[3\right]\left(k_{3}-1\right),$$

where $k_{3}=t_{3}/\Delta t_{S}$ represents the initial discrete time step of the third model phase.

The variance ($P_{3}^{A}\left[2,2\right]\left(k_{3}\right)$) of this initial condition is obtained by propagating the variances of $x_{2}^{A}\left[2\right]\left(k_{3}-1\right)$ and $x_{2}^{A}\left[3\right]\left(k_{3}-1\right)$ through (60).

Consequently the initial values of the assimilated state variables and their covariance matrix are defined as follows:

(62) $$x_{3}^{A}\left(k_{3}\right)=\left[\begin{array}{c} x_{2}^{A}\left[1\right]\left(k_{3}-1\right)\\ \frac{x_{2}^{A}\left[2\right]\left(k_{3}-1\right)x_{2}^{A}\left[3\right]{\left(k_{3}-1\right)}^{1.5}}{d}\\ x_{2}^{A}\left[3\right]\left(k_{3}-1\right)\\ x_{2}^{A}\left[4\right]\left(k_{3}-1\right)\\ x_{2}^{A}\left[5\right]\left(k_{3}-1\right)\\ 5.0\times 10^{-4}\;K/W\\ 2\;cm\\ 5.00\times 10^{-4}\;m^{0.5}/\mathrm{sA}\\ x_{2}^{A}\left[6\right]\left(k_{3}-1\right)\\ x_{2}^{A}\left[7\right]\left(k_{3}-1\right) \end{array}\right],\;P_{3}^{A}\left(k_{3}\right)=\mathrm{diag}\left\{\left[\begin{array}{c} P_{2}^{A}\left[1,1\right]\left(k_{3}-1\right)\\ P_{3}^{A}\left[2,2\right]\left(k_{3}\right)\\ P_{2}^{A}\left[3,3\right]\left(k_{3}-1\right)\\ P_{2}^{A}\left[4,4\right]\left(k_{3}-1\right)\\ P_{2}^{A}\left[5,5\right]\left(k_{3}-1\right)\\ 4\times 10^{-10}\;K^{2}/W^{2}\\ 16\;mm^{2}\\ 1\times 10^{-12}\;m^{0.25}/s^{2}A^{2}\\ P_{2}^{A}\left[6,6\right]\left(k_{3}-1\right)\\ P_{2}^{A}\left[7,7\right]\left(k_{3}-1\right) \end{array}\right]\right\}$$

As previously mentioned, the variance ($P_{3}^{A}\left[2,2\right]\left(k_{3}\right)$) is derived by propagating the variances of the assimilated states from phase 2 ($x_{2}^{A}\left[2\right]\left(k_{3}-1\right)$ and $x_{2}^{A}\left[3\right]\left(k_{3}-1\right)$) through (60).

#### 5.2.4. Phase 4: Contacts Opened—Arc Inside Splitter Plates

During the fourth arc model phase, the AACB’s internal inductance remains constant, as the arc is assumed to be inside the splitter plates, which are fully saturated. The parameters influencing measurement and model uncertainties in this phase are expressed by

$$\pi_{4}=\left[\begin{array}{cccc} v_{N} & L_{N} & R_{N} & L \end{array}\right],\;\xi_{4}=\left[\begin{array}{ccccccc} v_{N} & L_{N} & R_{N} & L & C_{P} & C_{A} & C_{S} \end{array}\right]$$

The values and standard deviations of $v_{N},\;L_{N}$ and $R_{N}$ are provided in Table 1. The values and standard deviations of the parameters for this phase, except for those related to the laboratory setup presented in Table 1, are provided in Table 4.

Considering the error sensitivity coefficients in the covariance propagation law (37) for this model phase, the primary contribution to the measurement model error covariance matrix, apart from (38), arises from the error affecting the arc inductance (*L*). However, this contribution remains negligible compared to the instrument-related contributions in (38). As a result, the measurement error covariance matrix is expressed as

(63) $$R_{4}\left(k\right)=R_{\epsilon^{\prime }}=\left[\begin{array}{cc} 4\times 10^{6}\;A^{2} & 0\\ 0 & 4\times 10^{2}\;V^{2} \end{array}\right]$$

Since this matrix remains constant over time, the measurement error covariance is considered stationary.

In the studied AACB, the thermal time constant ($R_{S}C_{S}$) is comparable to the duration of the fourth phase model. This is due to the significantly larger surface area where the external air interacts with the splitter plates compared to the rails and surrounding environment, preventing adiabatic conditions. Consequently, all state variables in (26) are taken into account.

In the studied AACB, the thermal time constant ($R_{S}C_{S}$) is comparable with the fourth phase duration. This is because the surface area where the external air contacts the splitter plates is significantly greater than that between the rails and environment, leading to the absence of adiabatic conditions. Therefore, all state variables in (26) are considered.

The error sensitivity coefficients in (41) indicate that the primary contributions to the error model arise from uncertainties in the external network voltage ($v_{N}$), inductance($L_{N}$) and resistance ($R_{N}$). Additionally, the error in the arc’s thermal capacitance ($C_{P}$) is the sole factor influencing the variance of the arc’s temperature rise error. Since this parameter is time-dependent, the model covariance matrix is not stationary.

Considering the propagation of the non-negligible uncertainties in the $\xi_{4}$ parameter, the resulting model error covariance matrix is expressed by

(64) $$Q_{4}\left(k\right)=\mathrm{diag}\left\{\left[\begin{array}{c} 3\times 10^{2}\;A^{2}\\ Q_{3}\left[2,2\right]\left(k_{4}-1\right)\\ \Delta t_{S}^{2}{\left(\frac{\partial f_{4}\left[3\right]\left(k\right)}{\partial \;C_{P}}\right)}^{2}\sigma_{C_{P}}^{2}\\ 1\;K^{2}\\ Q_{3}\left[5,5\right]\left(k_{4}-1\right)\\ Q_{3}\left[6,6\right]\left(k_{4}-1\right)\\ 1\;K^{2}\\ Q_{3}\left[10,10\right]\left(k_{4}-1\right)\\ 2\times 10^{-14}\;K^{2}/W^{2}\\ 5\;V^{2} \end{array}\right]\right\}$$

In (64), the variance of the root voltage (w(k)) is determined empirically. Given the number of splitter plates in the AACB under test, this voltage is limited to approximately (600 V).

The initialization of the assimilated state variables and their covariance matrix, as defined in (25), is performed using the current (*i*), the arc resistance constant ($k_{R}$), the temperature rises ($T_{P},\;T_{A}$, and $T_{S}$) and the thermal resistances ($R_{P-A},\;R_{A}$ and $R_{P-S}$). This ensures continuity with the previous phase. Specifically, continuity in the temperature rise of the splitter plates and the thermal resistance between the arc and splitter plates is maintained by aligning them with the temperature rise of the rails and the thermal resistance between the arc and rails from the preceding phase. However, the thermal resistance ($R_{S}$) could not be defined with continuity from the previous state. Therefore, its value was empirically assigned as 0.10(1) mW/K, accounting for the cooling effect of the external environment on the splitter plates.

The initialization of the assimilated state variables and their covariance matrix is expressed by

(65) $$x_{4}^{A}\left(k_{4}\right)=\left[\begin{array}{c} x_{3}^{A}\left[1\right]\left(k_{4}-1\right)\\ x_{3}^{A}\left[2\right]\left(k_{4}-1\right)\\ x_{3}^{A}\left[3\right]\left(k_{4}-1\right)\\ x_{3}^{A}\left[4\right]\left(k_{4}-1\right)\\ x_{3}^{A}\left[5\right]\left(k_{4}-1\right)\\ x_{3}^{A}\left[6\right]\left(k_{4}-1\right)\\ x_{3}^{A}\left[9\right]\left(k_{4}-1\right)\\ x_{3}^{A}\left[10\right]\left(k_{4}-1\right)\\ 1.0\times 10^{-4}\;K/W\\ E \end{array}\right],\;P_{4}^{A}\left(k_{4}\right)=\mathrm{diag}\left\{\left[\begin{array}{c} P_{3}^{A}\left[1,1\right]\left(k_{4}-1\right)\\ P_{3}^{A}\left[2,2\right]\left(k_{4}-1\right)\\ P_{3}^{A}\left[3,3\right]\left(k_{4}-1\right)\\ P_{3}^{A}\left[4,4\right]\left(k_{4}-1\right)\\ P_{3}^{A}\left[5,5\right]\left(k_{4}-1\right)\\ P_{3}^{A}\left[6,6\right]\left(k_{4}-1\right)\\ P_{3}^{A}\left[9,9\right]\left(k_{4}-1\right)\\ P_{3}^{A}\left[10,10\right]\left(k_{4}-1\right)\\ 4\times 10^{-14}\;K^{2}/W^{2}\\ 3\times 10^{1}\;V^{2} \end{array}\right]\right\}$$

### 5.3. Estimation Results

**Figure 12:**
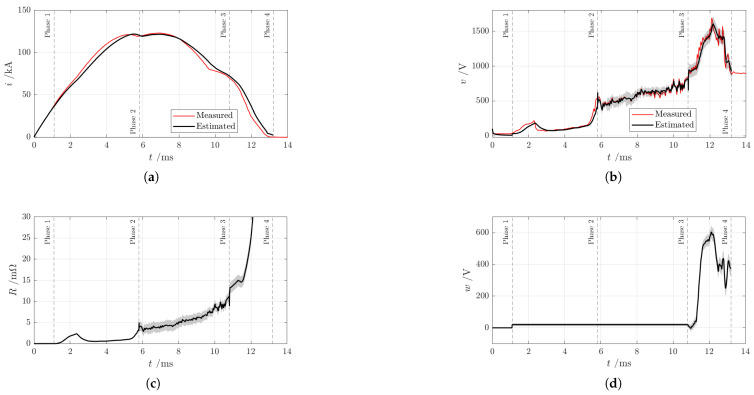

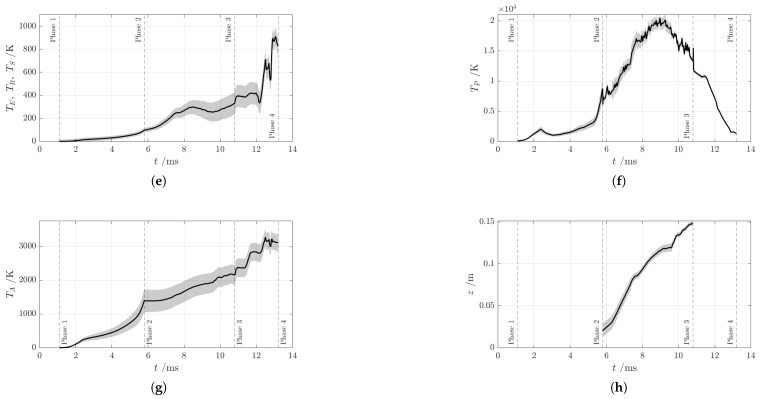
Estimation of the state variables. (**a**) Current through the test AACB. (**b**) Voltage between AACB’s contacts. (**c**) Arc resistance. (**d**) Arc root voltage. (**e**) Temperature rises of electrodes, rails and splitter plates. (**f**) Arc temperature rise. (**g**) Air temperature rise. (**h**) Arc position.

Some of the state estimates are shown in Figure 12. The reported error bands have a width of two standard deviations. Certain state variables are present only in specific phases. For example, the arc position is included only after the arc-on-rails-phase (contacts opened), as shown in Figure 12h.

Whenever possible, the general condition of continuity was enforced for all state variables between consecutive phases.

The current and voltage estimates in Figure 12a,b show good agreement with the measurements.

The arc resistance in Figure 12c exhibits a behavior similar to the voltage trend shown in Figure 12b and discussed in Section 5.1. During the first three model phases, the resistance increases at a lower rate, followed by a rapid rise when the arc reaches the area of the splitter plates. A similar pattern is observed in the interruption of AC currents within SF6 extinction chambers.

This process can be divided into two distinct stages. The initial stage is characterized by a gradual increase in resistance and is strongly influenced by the specific characteristics of the extinction chamber. The subsequent stage is much more rapid, with arc behavior largely determined by its evolution during the preceding stage. These observations form the foundation of the well-known KEMA black-box arc model [3,30].

In the proposed model, arc resistance increases up to 15 mΩ during the initial stage and reaches approximately 200 mΩ in the rapid stage.

In Figure 12d, the arc voltage of the arc roots (*w*) rises sharply toward the final model phase, reaching its maximum expected value. This increase is driven by the rapid penetration of the arc into the splitter plates, which causes a significant temperature rise in the plates, as shown in Figure 12e. Furthermore, this rapid penetration is expected to cool the arc to the point of extinction, leading to current interruption and setting the voltage across the AACB contacts equal to the external network voltage.

Estimations of arc temperature rise in Figure 12f indicate a peak temperature of approximately 20,000 K during the rail model phase, while the air temperature rise in Figure 12g reaches about 3000 K in the final model phase. These high temperatures are consistent with values reported in similar studies [31,32] and with MHD simulations conducted on the AACB under test. However, slight discontinuities are observed during the transitions between model phases 2 and 3, as well as between model phases 3 and 4. These discontinuities result from the model switching between different phases. Another noticeable feature is the increasing uncertainty in temperature rise during model phase 2. This uncertainty stems from the non-observability of the thermal network proposed in Figure 7b, due to the lack of correlation between arc resistance and arc temperature, which is characteristic of this model phase. Consequently, this growing uncertainty affects the estimation of states and their associated errors in the subsequent model phases.

The arc position shown in Figure 12h demonstrates a steady progression toward the splitter plates, with no evidence of reversal. This behavior is supported by the estimated AACB voltage presented in Figure 12b, which shows no indication of back-ignition or arc re-strike near the contact region. These results are consistent with the expected arc trajectory.

The average arc propagation speed is estimated to be approximately 25 m/s, consistent with the values reported by Lisnyak [18] for low-current conditions (below 1000 A) and constant rail separation. As the arc elongates during its motion, the resulting increase in arc resistance contributes to current limitation. At the same time, the extended arc path enhances heat dissipation to nearby components within the interruption chamber. However, this also tends to moderate the rate of increase in plasma resistance due to more effective thermal diffusion.

During phase 3, pronounced voltage oscillations are observed, which are also reflected in the arc temperature and resistance estimates, though not in the arc position. As discussed in [33], such oscillations may be attributed to continuous, small-scale back-commutations of the arc between the rails and the central region of the splitter plates. This suggests that modeling the arc as a rigid column oversimplifies its dynamic behavior. Nonetheless, this assumption remains adequate for capturing the dominant features relevant to the arc’s progression and its impact on circuit performance.

It is also noteworthy that the peak arc temperature occurs with a delay relative to the peak current. While a direct proportionality might be expected due to the limited thermal capacity of the arc column itself, this temporal offset suggests the involvement of additional thermal energy storage elements within the interruption chamber. Consequently, after current interruption, these components are expected to cool with a characteristic time constant governed by their thermal properties.

## 6. Conclusions

This paper presents a highly innovative tool for non-invasive condition monitoring of AACBs. This tool employs voltage and current measurements at the AACB terminals, incorporating them into a multi-parameter lumped model of the electrical arc within the AACB. This approach offers several key advantages. It enables rapid estimation of critical physical parameters that characterize AACB behavior while maintaining minimal computational overhead. Unlike more complex arc models that require extensive calibration of numerous physical parameters, such as those solving the magneto-hydro-dynamic equations, this method provides immediate, interpretable results. Furthermore, unlike black-box models of general circuit breakers, this model offers direct insights into the physical behavior of specific AACBs, enhancing both accuracy and diagnostic capabilities.

Several extensions of the proposed approach can be explored to further enhance its capabilities, including the following:

- Enhanced arc model description, including improved representation of arc behavior, the incorporation of mechanical contact separation dynamics, the possibility of the arc returning to the rails and a more detailed modeling of heat transfer, including radiation effects;
- Implementation of Bayesian automatic model selection to define arc model phases, replacing manual voltage signal analysis by an expert;
- Analysis of the full O-CO-CO-CO sequence instead of analyzing individual CO events one at a time, which would allow the cumulative effects of previous tests on subsequent tests to be considered;
- Improvement of the accuracy of the lumped-parameter model through a calibration phase tailored to specific AACB types or even individual devices, which can be achieved using distributed parameter simulations for AACB-type calibration and/or simple experimental tests on specific units (for example, thermal constants can be determined through low-current heating experiments followed by cooling-phase observations);
- Incorporation of additional external measurements during standard short-circuit tests, such as the power supply voltage ($v_{N}\left(t\right)$) and the external case temperature. These additional measurands can help refine the accuracy of extended Kalman filter estimates and reduce uncertainty.

By pursuing these enhancements, the proposed approach can be further developed into a more robust and more accurate condition monitoring tool.

## Figures and Tables

**Table 1:** Laboratory setup parameters and standard deviations present during all phases.

Quantity	Magnitude	Standard Deviation Symbol
$v_{N}$	0.92(5) kV	$\sigma_{v_{N}}$
$R_{N}$	5.0(3) m Ω	$\sigma_{R_{N}}$
$L_{N}$	24.4(4) μ H	$\sigma_{L_{N}}$

**Table 2:** Parameters and standard deviations present during contact opening (phase 2; See Figure 7a,b).

Quantity	Magnitude	Standard Deviation Symbol
*L*	0.4(1) μ H	$\sigma_{L_{2}}$
*E*	20(5) V	$\sigma_{E}$
$C_{P}$	0.20(1) J/K	$\sigma_{C_{P}}$
$C_{A}$	5.0(3) J/K	$\sigma_{C_{A}}$
$C_{E}$	0.45(3) kJ/K	$\sigma_{C_{E}}$

**Table 3:** Parameters and standard deviations present during the arc-on-rails phase (see Figure 8a and Figure 9a,b).

Quantity	Magnitude	Standard Deviation Symbol
*L*	0.41(8) μH–0.71(8) μH	$\sigma_{L}$
∂L/∂i	0.5(1) nH/kA–0.9(2) nH/kA	$\sigma_{\partial L/\partial i}$
*E*	25(5) V	$\sigma_{E}$
$C_{P}$	0.20(1) J/K	$\sigma_{C_{P}}$
$C_{A}$	0.10(1) kJ/K	$\sigma_{C_{A}}$
$C_{R}$	0.45(3) kJ/K	$\sigma_{C_{E}}$
*ℓ*	5(4) cm to 40(4) cm	$\sigma_{l}$

**Table 4:** Parameters and standard deviations present during the arc-inside-splitter-plates phase (see Figure 10a,b).

Quantity	Magnitude	Standard Deviation Symbol
$C_{P}$	0.20(1) J/K	$\sigma_{C_{P}}$
$C_{A}$	0.10(1) kJ/K	$\sigma_{C_{A}}$
$C_{S}$	2.5(1) kJ/K	$\sigma_{C_{S}}$
*L*	0.71(9)μH	$\sigma_{L}$

## Data Availability

Data are contained within the article. The original contributions presented in the study are included in the article. Further inquiries can be directed to the corresponding author.

## Abbreviations

The following abbreviations are used in this manuscript:

AACB	Air Arc-chute Circuit Breaker
SP	Splitter Plate
MHD	Magneto-Hydro-Dynamic
FEM	Finite-Element Method
